# Modification of starch synthesis in food crops using CRISPR/Cas9 gene editing technology for changing climate

**DOI:** 10.1007/s44154-025-00278-x

**Published:** 2026-01-07

**Authors:** Liangjie Niu, Hui Liu, Nannan Wang, Xiaolin Wu, Fuju Tai, Xiuli Hu, Wei Wang

**Affiliations:** 1 Dabie Mountain Laboratory, Henan Key Laboratory of Tea Plant Biology, College of Tea and Food Science, Xinyang Normal University, Xinyang, 464000 China; 2 Key Laboratory of High-Efficiency Production of Wheat-Maize Double Cropping, College of Life Sciences, Henan Agricultural University, Zhengzhou, 450046 China

**Keywords:** Starch modification, Food crops, Climate change

## Abstract

Starch is a principal storage component in plants, significantly influencing the yield and quality traits of major crops. Climate change, particularly drought and high temperatures, severely affects starch biosynthesis in crops, leading to reduced starch yield and quality. The composition and properties of native starch, such as its low amylose content, substantially affect its nutritional value and industrial applications. To tackle these issues, genes coding for starch synthetic enzymes or those involved in the regulation of starch biosynthesis could be targeted for site-directed mutation to improve starch traits in crops. The application of gene editing technology in crops, notably CRISPR/Cas9, has facilitated the precise manipulation of starch biosynthesis. This review summarizes current knowledge on the biosynthesis and regulation of starch and the influence of climate change on these processes. It highlights advancements in modifying starch biosynthesis in food crops using CRISPR/Cas9. We discuss the strategy of improving starch traits and stress tolerance in response to climate change challenges and propose future directions for research on starch modification in food crops. Developing climate resilient crops capable of stable starch production is crucial for ensuring food security in the face of a changing global climate and an increasing world population.

**Graphical Abstract:**

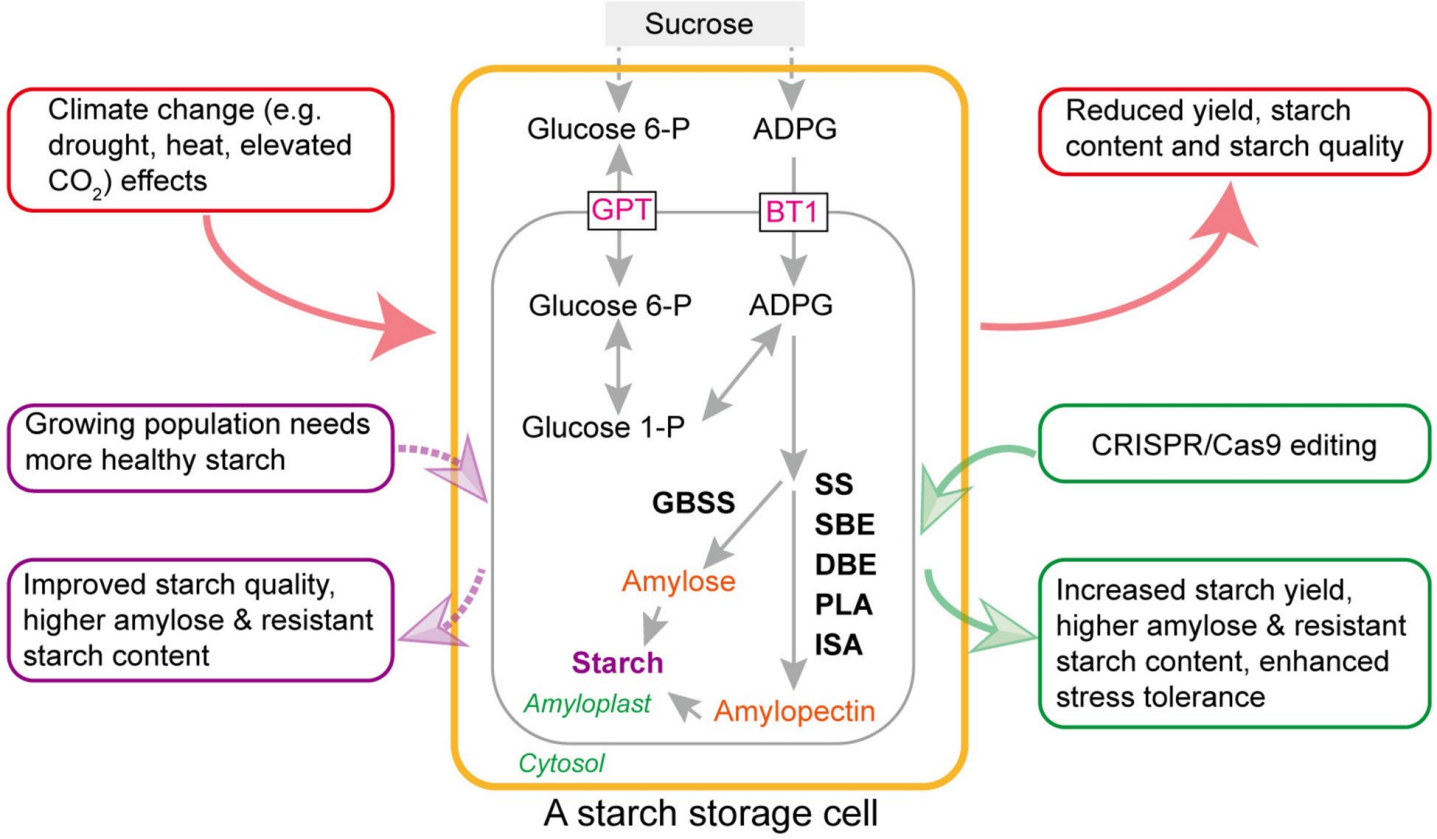

## Introduction

Starch is the primary carbon reserve in plants and the second most abundant renewable biopolymer on Earth, following cellulose (Li et al. 2023a). Globally, cereals (e.g., wheat, rice, and maize) and starchy tuber and root crops (e.g., potato and cassava) are the primary sources of starch in human diets. Starch is composed of α−1,4-linked glucose units (amylose) that are intermittently branched with α−1,6-linkages (amylopectin), existing in semi-crystalline, insoluble starch granules (SG) (Smith and Zeeman 2020). The composition, structure, and morphology of SG vary significantly among plant species and the organelles in which they are deposited and depend on growth conditions (Yu et al. 2017; Cuesta-Seijo et al. 2019; Smith and Zeeman 2020; Niu et al. 2019a). Starch is synthesized in plastids—chloroplasts in leaves and amyloplasts in non-photosynthetic organs (e.g., seeds, stems, and roots). Leaf starch, also known as transitory starch, is produced directly from photosynthesis during the day and degraded at night (Smith and Zeeman 2020; Niu et al. 2024). Storage starch in seeds, tubers, and roots is maintained over longer periods and is predominantly utilized as food and feed, a renewable resource for bioenergy, and industrial raw materials (Li et al. 2023a; Niu et al. 2023). This review focuses on storage starch.

The continuing growth of the global population is a rigorous challenge for crop starch production, with the increased demand for healthier starch-based foods. For example, a diet rich in resistant starch (RS), which is tolerant to amylase hydrolysis and is hardly digested in the small intestine, can help control blood sugar levels in diabetic patients (Huang and Liu 2023). The RS content in crops is positively correlated with the amylose content (Guo et al. 2023) and is also determined by the intrinsic properties of starch, such as the amylose-to-amylopectin ratio, fine structure, and SG morphology (Shen et al. 2022). The amylose-to-amylopectin ratio is especially critical to the nutritional quality and functional properties of starch and its end uses (Li et al. 2023a). Cereals with high amylose and RS content are increasingly valued for their potential to improve human health and lower the risk of serious noninfectious diseases (Shen et al. 2022). However, the contents of amylose and RS are usually low in cereals, such as wheat and rice. In potato tubers, starch is composed of 80% amylopectin and 20% amylose (Brummell et al. 2015). Thus, there is a growing need to develop cereals with high amylose/RS content to meet public health and nutritional requirements.

Global climate change poses an unprecedented challenge to global food supplies in the twenty-first century. Climate change—primarily characterized by drought and high temperatures—is reducing the global production of economically important crops, such as wheat, rice, and maize (Steinwand and Ronald 2020). Most cultivated crops are not tolerant of extreme climatic environments. The quantity and quality of starch in cereal grains are particularly vulnerable to climate changes, which often cause detrimental changes in amylose content, starch structure, and functional properties (Wu et al. 2022; Zhao et al. 2022; Zhang et al. 2022). Under the most severe climate change scenario and without adaptation, simulated crop yield losses are projected to range from 7 to 23% (Rezaei et al. 2023). The loss of starch would account for the vast majority of the yield losses, as starch constitutes approximately 70% of the dry weight (w/w) of grains in starch crops (Jung et al. 2008). Taking wheat as an example, elevated temperatures predominantly influence the accumulation of starch within wheat grains, with about 40% of the variability in annual wheat production being attributed to heatwave and drought stress (Mao et al. 2023). Furthermore, the nutritional quality of C_3_ cereals is adversely impacted by severe climate change, and heatwave reduces starch content but increases grain protein and mineral contents (Ben Mariem et al. 2021). It is estimated that food production must double by 2050 to feed a projected population of 9.7 billion (Godfray et al. 2010). Therefore, the global demand for high-yield starchy crops and starch-based products is more pressing than ever in the context of climate change. Therefore, enhancing climate resilience and improving the starch biosynthesis of crops in a changing climate is an urgent goal of crop science research and crop breeding.

The world food supply depends heavily on an increase in grain yield, which is primarily determined by starch accumulation in crops. To meet future food challenges, future crops should have a high RS content for human health (improved starchy crops) and be resistant to environmental stresses (climate change-ready crops). The pivotal role of starch in food, nutrition, and industrial applications has driven the diversification of crops with desirable starch traits. In recent years, the application of gene editing technologies, particularly CRISPR/Cas9, has significantly advanced the rational manipulation of starch biosynthesis and starch quality in crops (Chen et al. 2021). This review briefly describes the starch biosynthesis pathway in crop plants, the impact of climate change on this pathway, and highlights the target enzymes for gene editing. We focus on the CRISPR/Cas9 technology application for modifying starch biosynthesis in crops because gene edited crops are preferred in the future due to their non-transgenic modification feature. We discuss the strategy of improving starch traits and stress tolerance in response to climate change challenges. Finally, we present perspectives on the development of climate-resilient crops with improved starch traits. Targeting the starch biosynthesis pathway to develop climate-resilient crops will greatly contribute to cleaner production and food security.

## Starch synthesis in cereal endosperms and the impact of climate change

### Core process and regulation of starch biosynthesis

**Fig. 1:**
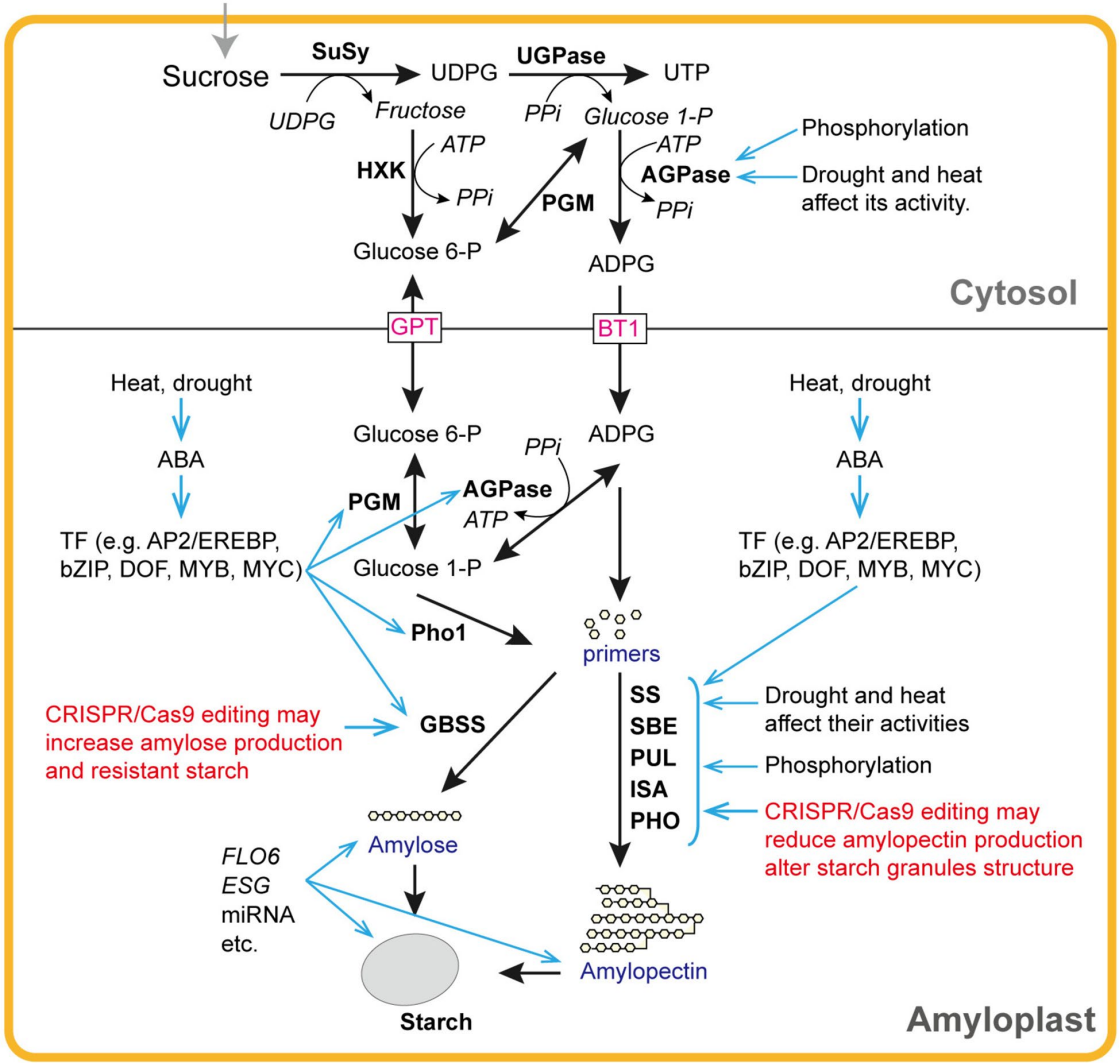
Simplified process of starch biosynthesis in a cereal endosperm cell. Key targets for CRISPR/Cas9 editing to modify starch synthesis are indicated. The adverse effects of climate change on the activities of key enzymes are highlighted. AGPase (EC 2.7.7.27) catalyzes the synthesis of ADP-glucose, which serves as an activated glycosyl donor for α−1,4-glucan synthesis. It is composed of two small subunits (SSU) and two large subunits (LSU), with SSU being primarily catalytic and LSU regulating responses to allosteric effectors. In cereal endosperms, cytosolic AGPase activity controls starch synthesis. AGPase is activated by 3-phosphoglyceric acid and inhibited by inorganic phosphate (Pi) through allosteric regulation. SS (EC 2.4.1.21) transfers the glucosyl moiety of ADP-glucose to the non-reducing end of a pre-existing α−1,4-linked glucan primer to synthesize amylose and amylopectin. SS is classified into five main groups: SSI–SSIV and GBSSI. GBSSI is responsible for amylose synthesis in cereal endosperms, and SSI-SSIII primarily elongates amylopectin. SBE (EC 2.4.1.18) catalyzes the formation of α−1,6-linkages in amylopectin. Two categories of SBEs—SBEI and SBEII—exist in multiple isoforms. Simultaneous suppression of SBEI and SBEII has been shown to increase resistant starch (RS) and amylose content and modify the amylopectin structure more significantly than suppression of SBEII alone (Utsumi et al. 2022). DBE (EC 3.2.1.68) is involved in amylopectin biosynthesis by hydrolyzing some α−1,6-linkages of pre-amylopectin chains. Two types of DBE, isoamylase (ISA, EC 3.2.1.68) and pullulanase (PUL, EC 3.2.1.41), differ in their substrate preferences

In cereal endosperms, storage starch is synthesized in specialized amyloplasts, and the core pathway of starch biosynthesis has recently been reviewed (e.g., Huang et al. 2021a; Liu et al. 2022a; Kang et al. 2023; Niu et al. 2023, 2019b), detailing the coordinated action of starch biosynthetic enzymes. These predominantly plastid-localized enzymes include ADP-glucose pyrophosphorylase (AGPase), soluble starch synthase (SS), granule-bound starch synthase (GBSS), starch branching enzymes (SBE), and debranching enzymes (DBE) (Fig. 1). Similar starch biosynthetic enzymes and multienzyme complexes have been identified in cassava storage roots (He et al. 2022).

AGPase catalyzes the conversion of glucose-1-phosphate (derived from sucrose transported from leaves) to ADP-glucose, which serves as a substrate for primer synthesis. ADP-glucose is utilized by GBSS and SS for the elongation of α−1,4-glucan chains, which are intermittently branched by SBE. GBSS specifically synthesizes amylose in the endosperm, directly influencing amylose levels, while SS is instrumental in elongating amylopectin chains and defining the amylopectin structure. Amylopectin synthesis involves the collaborative action of SS, SBE, and DBE, with SBE catalyzing the formation of α−1,6-glucosidic linkages and DBE removing excess or improper glucan branches. These core enzymes are significant targets of genome editing for manipulating starch biosynthesis.

Starch biosynthetic enzymes are highly conserved among plant species, suggesting a common evolutionary origin. They exist in multiple isoforms, particularly in cereals, and contribute variably to starch formation (Wang et al. 2023a). For instance, at least eight distinct SS isoforms exist in rice—SSI, SSIIa, SSIIb, SSIIc, SSIIIa, SSIIIb, SSIVa, and SSIVb—of which SSI, SSIIa, and SSIIIa are predominantly responsible for the amylopectin biosynthesis in endosperm (Fujita et al. 2006). This diversity, resulting from ancient genome duplication and subsequent amino acid deletions or mutations at active sites, presents a challenge for gene editing aimed at modifying specific isoforms (Qu et al. 2018). This inevitably increases the difficulty of gene editing for making changes to certain isoforms.

Many regulatory factors influencing starch biosynthesis in plants have been identified (Table 1, Fig. 1). For example, transcription factors (TF) such as NAC, bZIP, MADS, and MYB positively regulate starch biosynthesis by upregulating starch synthesis-related genes (SSRG), such as *GBSSIa* (*Wx*), *SSI*, *SSII*, and sucrose synthase gene *SuSy* in wheat (Kumar et al. 2018; Gao et al. 2021), rice (Liu et al. 2022b), and maize (Dong et al. 2019; Zhang et al. 2019a). In sorghum, *SbDof21* binds to and transactivates *GBSSI*, thus regulating starch biosynthesis and potentially enhancing the starch content (Xiao et al. 2022). 14–3-3 proteins may regulate starch accumulation by phosphorylating BEII, SSI, and SSII in developing barley grains (Chen et al. 2021). *ENLARGED STARCH GRAIN1* (*ESG1*) encodes a protein localized in chloroplast and amyloplast membranes, and its mutation leads to defective galactolipid synthesis, reduced starch content, altered starch properties, and abnormally enlarged SG in rice (Wang et al. 2021a).

**Table 1:** Examples of potential factors regulating starch biosynthesis in cereal grains

Targets	Function	Crop	Reference
lncRNA TCONS_00130663	Regulating RS biosynthesis, strong negative correlation with *SBEIIb*	Wheat	Madhawan et al. 2020
Ten RING protein genes	Enhances amylose synthesis and associates with GBSSI and SBEIIa	Wheat	Parveen et al. 2021
*NAM-B1*	Negatively regulating of flag leaf senescence caused by drought	Wheat	Borrill et al. 2015
SNPs in *SSI* and *SSIIIA*	Related to low glycaemic index of rice varieties	Rice	Priya et al. 2021
Plastidial disproportionating enzyme1 (*DPE1*)	Transferring maltooligosyl groups from amylose and amylopectin to amylopectin in endosperm	Rice	Dong et al. 2015
*ENLARGED STARCH GRAIN1*	Affects amyloplast development and starch synthesis in rice endosperm	Rice	Wang et al. 2021a
*OsAT1 (*encoding an anion transporter)	Negatively regulates grain size and yield; significantly increases starch in RNAi *OsAT1* lines	Rice	Liu et al. 2022c
*GWD1*	Enhances starch yield and quality, seed germination, and drought tolerance	Rice	Wang et al. 2021b
*Isoamylase 1 (ISA1)*	Inhibition of its expression leads to abnormal SG, decreased amylose and total starch content	Rice	Chao et al. 2019
*Sucrose synthase (SuSy)*	Overexpression of *ZmSUS1* increases the amylose content in maize endosperm	Maize	Li et al. 2023b
*ZmMADS1 (a TF)*	Up-regulating starch synthesis-related genes; overexpression increases amylose and total starch content	Maize	Dong et al. 2019
*ZmNAC128, ZmNAC130*	Both TF coordinate the accumulation of starch and protein in endosperms	Maize	Zhang et al. 2019a
6-phosphogluconate dehydrogenase	Mitigates grain yield losses in high temperatures by increasing kernel number	Maize	Ribeiro et al. 2020
*Dof21 (a TF)*	Bind and transactivate GBSSI	Sorghum	Xiao et al. 2022

Post-translational modifications of starch biosynthetic enzymes, such as phosphorylation of SBE and DBE by the thioredoxin/ferredoxin system, facilitate rapid responses to environmental factors (Tappiban et al. 2021). A study revealed that serine 31 phosphorylation of the AGPase large subunit drives the regulation of AGPase activity in maize, and serine 31 mutations appear to enhance AGPase activity, thus providing the potential for targeted improvements of AGPase activity for increased starch content (Yu et al. 2023). Starch phosphorylase (PHO) is crucial for metabolizing sucrose into carbon precursors essential for starch biosynthesis in maize endosperm (You et al. 2020). Additionally, 19 miRNAs modulate starch biosynthesis in maize endosperm by regulating the expression of 19 target genes (Zhang et al. 2019b). Long non-coding RNAs are also involved in regulating RS synthesis in bread wheat (Madhawan et al. 2020).

Finally, analysis of mutations or nucleotide polymorphisms in relevant genes has revealed proteins that influence SG size and morphology. Overexpression of *SS1* significantly increases SG size and starch content, particularly amylopectin, in transgenic sweet potato (*Ipomoea batatas*) (Wang et al. 2019). A carbohydrate-binding protein, B-GRANULE CONTENT 1 (BGC1), regulates B-type SG initiation in wheat and barley, with its effects varying with gene dosage and developmental stage (Chia et al. 2020). The mutation of *Floury Endosperm 6* (*FLO6*) results in fractured SG in barley (Saito et al. 2018) and smaller, irregular SG compounds in rice (Peng et al. 2014). Changes in SG morphology, such as the transition from polyhedral to spherical granules in rice, have been linked to mutations in *SS3a*/*SS4b* (Toyosawa et al. 2016). Similarly, mutations in *SS4* in Arabidopsis alter granules from discoid to spherical (Malinova et al. 2017).

The current understanding of the key enzymes, proteins, and regulatory factors involved in starch biosynthesis provides clues to starch modification in food crops through gene editing technology. However, many aspects of the regulatory network governing starch biosynthesis need to be elucidated.

### Climate change’s impact on starch biosynthesis

Environmental factors associated with global climate change, especially drought and heat, significantly affect cereal yield and grain quality (Senapati et al. 2019). Starch biosynthesis is particularly sensitive to heat and drought compared to storage proteins (Kim and Kim 2021). During the reproductive and grain-filling phases, drought, and heat, especially their combination, compromise the leaf capacity of stressed crops to produce photosynthates for developing grains, and suppress the activities of starch biosynthetic enzymes, leading to reduced starch content and altered SG structure in mature grains (Lu et al. 2019) (Fig. 1).

Drought during the flowering stage causes a significant reduction in total starch and amylose content in cereal grains (Yang et al. 2019; Wu et al. 2022). In maize, prolonged soil drought impairs amylopectin synthesis but increases the degree of polymerization (DP) of amylopectin (Wu et al. 2022). In wheat, drought reduces grain starch content by 10.43% but increases gluten content by 15.75%. In particular, drought alters the SG structure and properties, amylose digestibility, size distribution, and branch chain length (Yu et al. 2016; Wu et al. 2022), affecting the consistency of final wheat and maize products. The adverse effect of drought on starch synthesis is related to the inhibition of starch biosynthetic enzyme activities. For example, the activities of AGPase, GBSS, SS, and SBE significantly decline in developing maize grains under post-silking soil water deficits (Yang et al. 2019). The decrease in AGPase activity leads to premature cessation of starch deposition, and the reduction in SS activity largely accounts for the decreased starch content in wheat grains (Kim and Kim 2021). Due to its high *K*_*m*_ (low affinity) for ADP-glucose relative to SS, a slight decrease in GBSS activity can significantly lower the amylose-to-amylopectin ratio, thus reducing the RS and amylose contents in grains (Figueroa et al. 2022). Additionally, activation of TFs such as AP2/EREBP, bZIP, MYC, and DOF by drought signals positively modulates starch biosynthesis in cereals (Wu et al. 2019; Kim and Kim 2021), which can be used for devising strategies for modulating starch production in resilient crops.

Global surface temperatures steadily increased from 1973 to 2022 (Samset et al. 2023). It is predicted that a 2.0 °C rise in average temperature would lead to a 20% to 40% decline in global crop yield (Fatima et al. 2020), and the loss would increase by another 5% when heat stress is coupled with drought (Lesk et al. 2021). Major cereals, such as wheat, rice, and maize, grow optimally within a temperature range of 20–30 °C. Heat stress negatively affects photosynthetic rate, starch synthesis, enzyme activity involved in starch synthesis, starch content, and composition and is mainly responsible for reduced starch yield in global cereal crops (Cuesta-Seijo et al. 2019). In wheat, temperatures exceeding 30 °C decrease the grain-filling rate, leading to reduced starch content and altered starch composition but increased storage protein content in grains (Zhao et al. 2022). In maize, heat stress occurring during the grain-filling period can cause reduced starch synthesis, kernel number, and grain yield, but causes an increase in the percentage of amylose and short-chain amylopectin in endosperm starch, resulting in poor SG structure (Lu et al. 2014). In rice, heat stress at the filling stage causes the formation of loosely packed SG, a reduction in grain weight and amylose content, but an increased occurrence of chalky and abnormal grains (Zhang et al. 2016, 2018a), altogether resulting in low yield and poor eating quality.

The detrimental impact of heat stress is also evident in the heat susceptibility of key enzymes and their genes involved in starch biosynthesis in temperate cereals (Lu et al. 2019). As the rate-limiting enzymes in starch biosynthesis, SS is sensitive to heat stress, with a strong activity at 22 °C in wheat (Zhao et al. 2022). SS becomes largely non-functional above 35 °C, greatly restricting starch synthesis in developing grains (Lu et al. 2019). The activities of GBSS, AGPase, and SBE are also heat-labile in rice and maize (Yang et al. 2018). The significantly reduced ratio of amylose to total starch in non-waxy rice endosperms is mainly due to the suppression of GBSS activity and *Wx* gene expression by heat stress at the filling stage (Goswami et al. 2022). Alterations in the glucan chain length under heat stress are primarily attributed to reduced SBE activity (Yang et al. 2018). Recently, a bZIP TF (OsbZIP60) was found to activate the expression of several starch/storage protein synthesis-related genes, particularly under heat stress (Cao et al. 2022).

As discussed above, climate change factors, primarily drought and heat, significantly influence starch biosynthesis and quality, particularly in C_3_ cereals (Fig. 1). A deeper understanding of how starch biosynthesis responds to climate change, combined with the strategic application of genome editing technologies, will facilitate the development of resilient crop varieties with stable starch synthesis in a changing climate.

## Modification of starch synthesis in food crops by gene editing technology

### The potential of CRISPR/Cas9 approaches for crop improvement

Crop breeding efforts have traditionally focused on improving grain yield and enhancing crop tolerance to various environmental stresses. Given the challenges posed by climate change, there is an urgent need for a faster breeding cycle (Xiong et al. 2022) to enhance crop resilience and productivity. Gene editing technologies, particularly CRISPR/Cas9, significantly accelerate the breeding process by reducing the time required to enhance agronomic traits and eliminating the need for crossing processes.

**Fig. 2:**
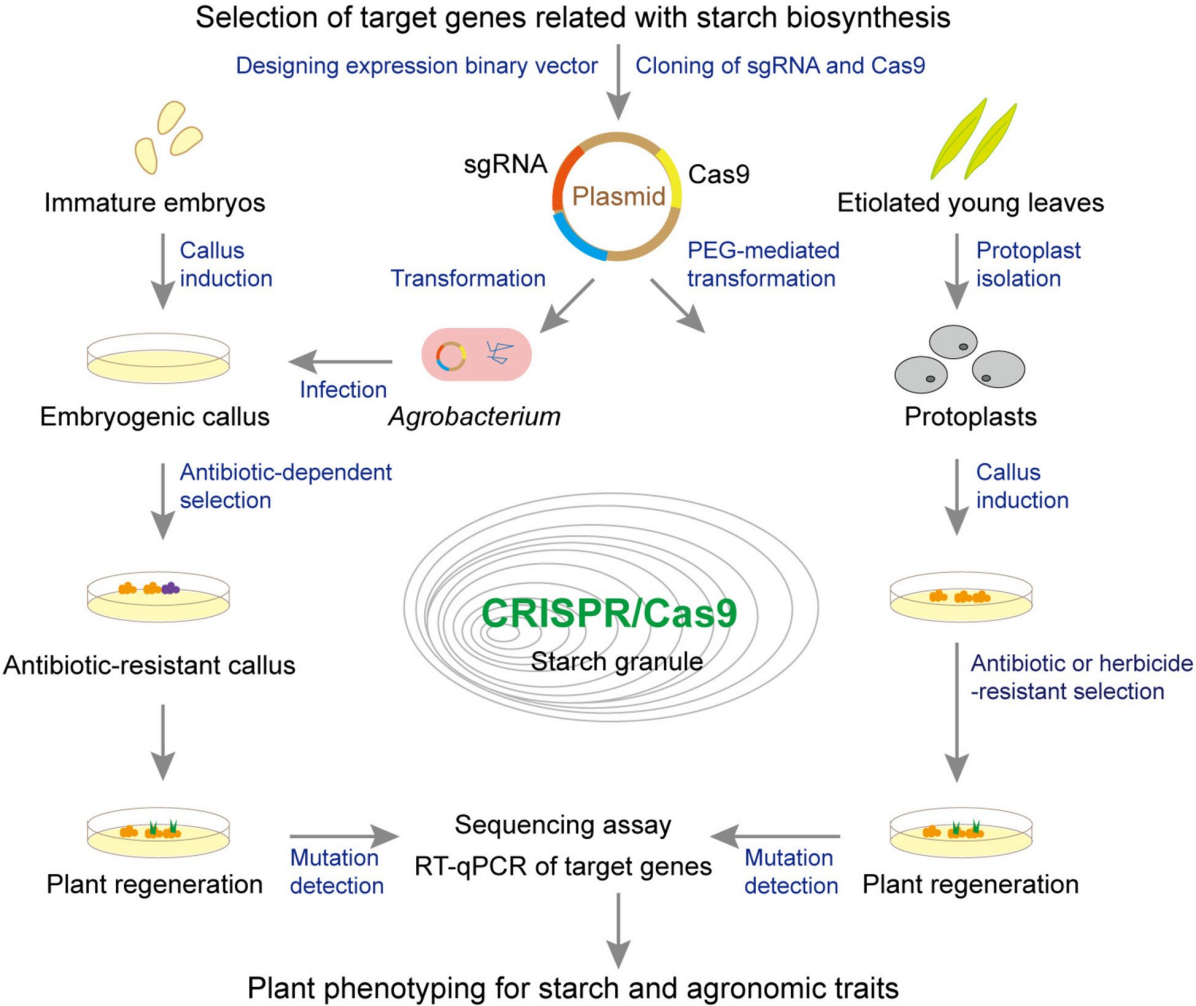
Simplified scheme of the main steps using CRISPR/Cas9 technology to edit target genes associated with starch biosynthesis in food crops

A typical CRISPR/Cas9 workflow involves the direct introduction of Cas9 and guide RNA (gRNA) expression cassettes into host protoplasts via genetic transformation (Fig. 2). Edited lines are regenerated from these gene-edited protoplasts, and homozygous mutants are identified through genotyping analysis. CRISPR/Cas9 technology allows for knockout, knock-in, base editing, and fine-tuning of gene expression (Chen et al. 2019) to silence undesirable genes or upregulate target genes by incorporating a few nucleotides in-frame (Razzaq et al. 2021). Gene redundancy in polyploid species may lead to off-target mutations, and several protocols have been developed to minimize these effects (Naeem et al. 2020). Moreover, various genome editing tools have been developed in recent years for targeted insertion and replacement in plants, such as (i) non-homologous end joining (NHEJ) repair-based DNA insertions/gene targeting, (ii) homology-directed repair-mediated gene insertions/gene targeting, (iii) transposon-based CRISPR/Cas-guided DNA insertions, and (iv) programmable recombinase/integrase-based editing (well-reviewed by Vollen et al. 2025).

CRISPR/Cas9 technology holds great potential for improving crop yield and quality by increasing beneficial nutrients or removing undesired compounds and enhancing crop tolerance to various environmental stresses. Introduced in 2013, CRISPR/Cas9 has become a popular choice because of its simplicity and high efficiency in plant genome editing (Shan et al. 2013). To date, CRISPR/Cas9 has been successfully applied in cereals such as wheat, rice, and maize (Zhou et al. 2014; Zhang et al. 2019c; Qi et al. 2020) and in starchy tuber and root crops such as potato (Tuncel et al. 2019) and sweet potato (Wang et al. 2019). As CRISPR/Cas9 does not typically involve the integration of exogenous DNA, it is considered a naturally occurring genome editing tool. Thus, the CRISPR/Cas9-edited crops are likely to be rapidly adopted and accepted in the field, thus contributing to improving global food supplies.

Since starch content in storage organs is a primary determinant of crop yield and quality, targeting the starch biosynthetic pathway is a practical approach to crop improvement. This can be achieved through the deactivation of genes with undesirable effects or the modification of their regulatory regions. The feasibility of this approach is supported by the identification of many core enzymes involved in starch biosynthesis and the availability of mutants with defects in starch synthesis. In the face of climate change, the development of edited crops that maintain stable starch synthesis is increasingly crucial. We next focus on the application of CRISPR/Cas9 in modifying starch biosynthesis, such as altering starch content, SG morphology (size), and fine structure in crop plants.

### Modification of starch content and quality by editing starch synthetic enzymes

Amylose and RS are beneficial for human health because they lower the risk of certain serious diseases. Cereal grains with high amylose content are a source of RS, and modified amylopectin with longer chain lengths also contributes to the formation of RS (Shen et al. 2022). Moreover, the amylose content in starchy crops affects not only the processing but also the application of starch in both the food and non-food industries (Wang et al. 2023b). Therefore, it is essential to modify starch biosynthesis by fine-tuning the amylose content and increasing the RS content in cereal grains. A viable method is the suppression of genes involved in amylopectin synthesis.

Starch synthesis in cereal relies greatly on the function of AGPase, GBSS directly determines amylose content, SS plays a critical role in the elongation of amylopectin chains, and SBE controls the synthesis of amylopectin and RS in crops. The high expression and strong activity of GBSS in starch storage organs are the basis for RS formation (Xu et al. 2021). Mutation or inhibition of the expression of *SS* and *SBE* in starch storage organs by CRISPR/Cas9 editing is the most well-known method for increasing amylose and RS content in crops. The fine-tuning of the gene expression of the GBSS gene (*Wx*) can control the amylose content within a moderate range. Through CRISPR/Cas9 editing of genes encoding starch biosynthetic enzymes, many crop plants with elevated amylose content or improved starch quality have been developed, particularly in wheat, rice, and potato (Table 2). Manipulating the genes encoding GBSS, SBE, and SS through CRISPR/Cas9 can significantly increase amylose content and improve RS content in crops such as wheat (Li et al. 2021), rice (Sun et al. 2017; Fu et al. 2023, 2022), barley (Yang et al. 2022), and potato (Andersson et al. 2018; Tuncel et al. 2019).

**Table 2:** Examples of CRISPR/Cas9 modification of starch traits in food crops

Species	Gene	Vector/Loss of function	Main effects on starch biosynthesis	Reference
Wheat	*SBEIIa*	pCXUN-Cas9/Knockout	Altered starch composition, structure and properties; significantly increased amylose, RS and protein contents in grains of triple-null lines	Li et al. 2021
Wheat	*Wx* (*GBSS*)	Knockout	Improved grain and starch quality	Zhang et al. 2021
Wheat	*NAC019*	pBUE411/Knockout	Reduced expression levels of genes involved in gluten and starch accumulation, reduced gluten and starch contents and flour quality in triple-null lines	Gao et al. 2021
Wheat	*bZIP28*	pOsU3-sgRNA/Knockout	Decreased starch content	Song et al. 2020
Rice	*Wx (GBSS)*	pJIT163-2NSCas9D10A/Knockout	Reduced amylose content, irregular SG shape, structure and aleurone layer	Zhang et al. 2018b; Pérez et al. 2019
Rice	*Wx promoter*	pGreenII 0800-LUC, pH-nCas9-PBE/Knockout	Fine-tuning the amylose content (significantly reduced) with improved grain quality	Xu et al. 2021; Huang et al. 2020; Zeng et al. 2020
Rice	*Wx*^*a*^*, Wx*^*b*^	Knockout	Much decreased amylose content (0.26 to 1.78%) in the *wx* mutant endosperms	Fu et al. 2022; Fu et al. 2023
Rice	*SSIIa, SSIIIa, SSIIIb*	pYLsgRNA-OsU6a/Knockout	Their loss of function coordinately confers high RS content (up to 10.8%) in rice	Wang et al. 2023b; Yang et al. 2025
Rice	*SBEI, SBEIIb*	pCXUN-Cas9/Knockout	*Suppression SBEI and/or SBEIIb* cause significantly increased amylose and RS content	Sun et al. 2017
Rice	*SSII-2, SSII-3*	pC1300-Cas9/Knockout	Decreased the amylose content in the absence of *SSII-2,* amylopectin structures of the *ssii-2 sii-3* similar to that in *SSII-2* RNAi lines	Huang et al. 2021b
Rice	*PHO*	VK005-01/Knockout	Homozygous T1 mutants displayed significantly decreased grain weight and quality, abnormal SG and amyloplasts, and decreased protein content but increased amylose and lipid contents	Liu et al. 2021
*Maize*	*Wx*	ZC01-DTM^wx^/Knockout	The average amylopectin content was 94.9%	Qi et al. 2020
Barley	*Wx*	pLGYE001/Knockout	Amylose and amylopectin contents of *waxy* mutants were zero and 31.73%, compared to 33.50% and 39.00%, respectively, in the wild-type	Li et al. 2024
Barley	*SSIIa*	pVK005/Knockout	Increased amylose content and substantial changes in transcriptome and metabolome	Yang et al. 2022
Potato	*SBE1, SBE2*	pAGM8031/Knockout	Various tuber starch phenotypes; knockout of *SBE2* alone primarily affects numbers of SG initiations; knockout both *SBEs* reduce amylopectin branching	Tuncel et al. 2019
Potato	*SBE3*	Knockout	Significantly reduced amylose content	Takeuchi et al. 2021
Potato	*Tubulin-like GTPase FtsZ1*	pKSE401/Knockout	Increased SG size by the formation of macro-plastids and altered the amylose/amylopectin ratio	Pfotenhauer et al. 2023
Sweet potato	*GBSSI, SBEII*	pCAMBIA1300/Knockout	Reduced amylose in *GBSSI*-knockout mutant, but increased amylose, fewer short chains and more long chains in *SBEII*-knockout mutant	Wang et al. 2019
Cassava	*GBSS*, *PROTEIN TARGETING TO STARCH* (*PTST1*)	pCas9-sgGBSS-FT; pCas9-sgPTST-FT/Knockout	Reduced or eliminated amylose content in root starch	Bull et al. 2018
Cassava	*SBE2*	pCAMBIA1301/Knockout	Increased amylose and RS content, reduced starch branching	Luo et al. 2022

The amylose content of cereals ranges from 0–30%, mainly depending on the presence of different *Wx* alleles, which is one of the major determinants of taste and cooking quality (Xu et al. 2021). The *Wx* gene in rice encodes GBSSI, which controls amylose synthesis in the endosperm (Xu et al. 2021; Fu et al. 2023, 2022; Zhou et al. 2016). Knocking out *Wx* in two elite japonica rice cultivars resulted in reduced amylose content in rice grains (Zhang et al. 2018b). By editing its coding sequences, *Wx* expression can be eliminated or reduced, allowing the amylose content in rice grains to be fine-tuned, thus producing glutinous rice with improved textures without affecting other desirable agronomic traits (Zhang et al. 2018b; Xu et al. 2021; Zhou et al. 2016). A moderate amylose content (15–25%) is a significant index for high-quality rice (Zhang et al. 2018a), which can be achieved by fine-tuning the expression of the target genes. Promoter editing using CRISPR/Cas9 allows quantitative regulation of *Wx* expression or the creation of novel *Wx* alleles with fine-tuned amylose levels, resulting in improved grain quality in rice (Huang et al. 2020; Zeng et al. 2020). This promoter editing does not damage *Wx* protein products and has few negative side effects on plant growth and development.

SBE changes the amylose-to-amylopectin ratio in starch by affecting the content of amylopectin. Obvious functional redundancy among SBE is observed in cereals as well as root and tuber crops, leading to synergistic effects in RS formation (Table 2). In wheat, through CRISPR/Cas9 editing of *SBEIIa* increases amylose and RS contents in grains (Li et al. 2021). However, the hexaploid nature of common wheat poses significant challenges for targeted gene mutations. By knocking out *SBEIIa* in winter and spring wheat cultivars, the *SBEIIa*-edited triple-null lines possess significantly increased amylose, RS, and protein contents in grains (Li et al. 2021). In rice seeds, two types of SSIII are predominantly expressed. The loss of function of *SSIIIa* and *SSIIIb* coordinately confers a high RS content (10.8%) in cooked rice (Wang et al. 2023b; Huang et al. 2024). Knocking out *SBEI* (Sun et al. 2017), and/or *SBEIIb* leads to significantly increased amylose and RS levels in edited rice, thus further affecting the quality of starch (Table 2). Currently, modification of starch synthesis in food crops was mainly conducted through CRISPR/Cas9 knockout of genes encoding starch biosynthetic enzymes or editing of their promoter sequences.

To date, CRISPR/Cas9 studies have targeted SSRGs that are highly expressed in starch storage organs, such as *Wx*, *SS*, and *SBE.* Isoamylase (ISA) functions by removing excess branches or improper branches, while pullulanase (PUL) is involved in starch degradation and probably in the trimming of pre-amylopectin chains during starch synthesis. Both are involved in amylopectin biosynthesis in endosperm and are crucial for the crystallization of starch. Thus, ISA and PUL may have the potential to increase RS content by editing their expression to alter the intrinsic properties of starch in cereal crops.

New root and tuber crops with high RS content have been created via *Wx, SSIII* and/or *SSII* mutation using CRISPR/Cas9. For instance, in potato and sweet potato, CRISPR/Cas9 has been applied to reduce amylose content while increasing amylopectin content (e.g., Wang et al. 2019; Takeuchi et al. 2021; Toinga-Villafuerte et al. 2022). Amylopectin-rich potatoes were developed by knocking out *GBSS* (Andersson et al. 2018). A novel potato starch with no detectable branches was produced by mutating eight potato *SBE* alleles (Xu et al. 2021). In sweet potato (allopolyploid, 2n = 6x = 90), *GBSSI* knockout reduced but *SBEII* knockout increased the amylose content in total starches in edited lines, thus altering the amylose-to-amylopectin ratio (Wang et al. 2019). Therefore, CRISPR/Cas9 can significantly improve starch qualities in polyploid plants through multicopy gene knockouts, contributing to the advancement of breeding in polyploid root crops.

Besides amylose and amylopectin, starch contains minor components, such as proteins and lipids (Wang et al. 2020), which bind to the surface of SG and affect starch digestion. The content of these components can be changed via CRISPR/Cas9. For example, gene editing was successfully used to disrupt approximately 30 α-gliadin genes in bread wheat, resulting in an 85% reduction in gluten in grains (Sánchez-León et al. 2018), suggesting that CRISPR/Cas9 can create a more suitable starch for bioethanol production by removing genes encoding proteins and oils in seeds.

These examples above indicate that starch biosynthesis can be modified by CRISPR/Cas9 editing SSRG for enhanced starch traits in crops. Many TFs, such as NAC, MADS, bZIP, and Dof, also regulate starch biosynthesis (Table 1) by regulating *cis*-acting elements in target genes. These TFs may be promising targets of CRISPR/Cas9 for producing edited crops with high quality and high nutrition.

### Modification of SG morphology and size

The morphology of SG is an important factor influencing starch functionality, such as in vitro digestibility, as enzymatic hydrolysis mainly occurs on the SG surface (Shen et al. 2022). SG size is negatively correlated with starch digestibility (Huang and Liu 2023). Small intact Ss with high crystallinity are resistant to amylolytic degradation. Moreover, SG size is a critical parameter for industrial processing (Li et al. 2023a). The modification of starch for versatile applications using genome editing has been reviewed in wheat (Lafiandra et al. 2022), potato (Del Mar Martínez-Prada et al. 2021), and sweet potato (Lyu et al. 2021).

CRISPR/Cas9 can create edited lines exhibiting altered SG morphology, size, and fine structure (Table 2). In wheat, knocking out *SBEIIa* increases shorter-chain amylopectin with a DP of 6–8 and larger chains > 18 DP while decreasing the proportion of DP 9–17 (Li et al. 2021), resulting in altered starch morphology, structure, and properties. In potato tubers, SG size correlates with the size of the plastids where they are produced. The division of plastids is significantly controlled by the tubulin-like GTPase FtsZ1 (Pfotenhauer et al. 2023). CRISPR/Cas9-edited *FtsZ1* potato lines show up to a 1.98-fold increase in SG size in tubers (Pfotenhauer et al. 2023). In barley, by knocking out *SSIIa*, SG size visibly reduced with increased amylose content and altered amylopectin composition (Yang et al. 2022).

### Modification of starch synthesis by editing non-starch synthetic enzymes

Several TFs and other proteins that influence starch amount and/or structure have been identified. NAC019-A1 negatively regulates starch synthesis in wheat endosperm (Liu et al. 2020; Gao et al. 2021). Triple knockout mutants of all three *TaNAC019* homeologs exhibited reduced transcript levels for genes involved in starch and gluten accumulation in wheat grains, leading to lower starch and gluten contents (Gao et al. 2021). TubZIP28, a bZIP family TF from *Triticum urartu*, and its homologue TabZIP28 from wheat, enhance starch synthesis in wheat (Song et al. 2020). In cassava, integration of the Arabidopsis* FLOWERING LOCUS T* gene in the genome-editing cassette accelerates flowering, and reduces or eliminates amylose content in root starch, thus speeding up ex situ breeding of *GBSS*- and *PTST1*-edited cassava (Bull et al. 2018). This novel, time-saving breeding technique could be applied to other crops to create novel varieties with improved traits for food and industrial applications.

Notably, the CRISPR/Cas9 system can be applied to study multigene families of starch biosynthetic enzymes and their dosage effects in rice (Huang et al. 2020; Xu et al. 2021), wheat (Li et al. 2021), and *Brassica napus* (Wang et al. 2022). For example, Li et al. (2021) generated a series of transgene-free mutant lines with either partial or triple-null *sbeIIa* alleles in both winter and spring wheat varieties, demonstrating that the effects of partial or triple-null alleles were dosage-dependent, with triple-null lines exhibiting more significant changes in starch composition, RS content, fine structures of amylopectin, and physiochemical and nutritional properties. Similarly, multiple sgRNA expression cassettes were assembled into a binary vector, and two rounds of transformation were employed to edit all six *SBE* genes in *Brassica napus*. As a result, five homozygous mutant lines carrying two to six mutations of *SBE* were obtained with an altered starch structure, allowing a comparison of the effects of editing different SBE isoforms (Wang et al. 2022). These CRISPR editing studies provide new clues to elucidate the distinct functions of isozymes and should facilitate the breeding of high RS crops to enhance human health.

## Improving both climate resilience and starch traits

To meet future food challenges, it is imperative to develop gene-edited crops with improved starch traits and enhanced climate resilience. Several case studies (discussed below) using RNAi and CRISPR/Cas9 have provided clues to achieve this goal.

### Case studies on enhancing climate resilience and starch synthesis

In rice, *OsMADS7*, a floral organ identity gene, is greatly induced by high temperatures at the early filling stage. RNAi suppression of *OsMADS7* in rice endosperm stabilizes the amylose content under heat stress (Zhang et al. 2018a). We expect new rice cultivars with high amylose content and thermotolerance to be created by CRISPR/Cas9 editing *OsMADS7*. In wheat, flag leaf senescence is a critical factor affecting starch traits in grains (Ren et al. 2023), which can be accelerated by drought and high temperatures (Senapati et al. 2019). Enhancing drought tolerance and delaying flag leaf senescence would improve wheat yield potential under climate change. By silencing the expression of *NAM-B1* (a TF) in wheat, NAM RNAi lines were shown to exhibit delayed leaf senescence (40% increased photosynthesis), without visible changes in starch content and grain weight, but the activity of SS in both transgenic and control genotypes was limiting for starch synthesis during the later filling stage (Borrill et al. 2015). This implies that delaying flag leaf senescence and improving SS activity could improve wheat yield potential under climate change. Clearly, for CRISPR/Cas9-edited *OsMADS7* and *NAM-B1* can be used to create new crop cultivars with enhanced climate resilience and improved starch traits. Moreover, CRISPR/Cas9 can overcome the shortcomings of RNAi approaches, such as the incomplete suppression of gene expression.

Glucan, water dikinase 1 (GWD1), is an essential enzyme involved in the first step of transitory starch degradation in leaves, but it also plays a key role in the seeds. *GWD1* overexpression lines showed a promotional effect on rice drought resistance, with multiple improved traits, including starch yield and quality and seed germination, whereas CRISPR/Cas9-edited lines were more sensitive to drought stress and salt stress (Wang et al. 2021b). Thus, upregulating the expression of *GWD1* by combining an appropriate promoter via CRISPR/Cas9 may enhance rice yield, starch quality, and climate resilience.

Starch synthesis in cereal relies greatly on the function of AGPase, but AGPase in cereal endosperms is heat-labile, leading to reduced amylose content and yield loss under heat stress (Yang et al. 2018). The AGPase in potato (*Solanum tuberosum*) tuber is heat-stable. Insertion of an N-terminal motif unique to heat-stable potato AGPase into recombinant maize endosperm AGPase greatly increases heat stability (a 70-fold increase in the half-life at 58 °C) (Linebarger et al. 2005). Therefore, a superior AGPase with increased heat stability would be beneficial to starch synthesis in cereal grains. Thus, new cereal cultivars with heat-stable AGPase and high thermotolerance can be generated via AGPase mutation by CRISPR/Cas9.

In maize, the pentose phosphate pathway in amyloplasts at the filling stage is heat-sensitive. By modifying the heat sensitive 6-phosphogluconate dehydrogenase, many kernels can develop under heat stress, indicating that this approach may enhance maize tolerance to global warming (Ribeiro et al. 2020). Further, transgenic potato plants overexpressing two thermostable genes—the CuZn-superoxide dismutase (*PaSOD*) gene from *Potentilla atrosanguinea* and the ascorbate peroxidase (*RaAPX*) gene from *Rheum austral*—exhibit enhanced starch biosynthesis and salt stress tolerance (Shafi et al. 2017). Starch content in transgenic plants was shown to be increased 2–threefold, with improved growth and reduced accumulation of reactive oxygen species (ROS) compared to the control plants (Shafi et al. 2017). SS in wheat is a promising target due to its extreme sensitivity to heat and significant role in controlling the flux of the starch synthesis pathway (Wang et al. 2018). Thus, enhancing the heat tolerance of starch synthetic genes or introducing thermostable genes would be a feasible strategy for high-quality crop breeding in a globally warming climate.

### CRISPR/Cas9 editing crop genotypes with improved starch traits or climate resilience

One strategy for increasing the climate resilience of crops via CRISPR/Cas9 is to alter genes that confer undesirable traits, such as susceptibility to drought and heat. Knocking out *SlMAPK3* in edited tomato plants resulted in enhanced heat tolerance by maintaining ROS homeostasis (Yu et al. 2019), and a loss of function of auxin response factor 4 enhanced edited tomato plants’ tolerance to salinity and osmotic stress (Bouzroud et al. 2020). Possibly, CRISPR/Cas9-driven knock-out of genes that negatively regulate stress tolerance in crop genotypes with improved starch traits would upgrade climate resilience and ensure stable starch production under environmental stress.

Tolerance to diverse abiotic stresses, such as drought and heat, exists within crop gene pools and elite germplasms. Moreover, conventional and transgenic breeding have produced a number of stress-resistant crops, some of which have been applied in production (Anwar and Kim 2020). New climate-resilient varieties could be created through further enhancements via CRISPR/Cas9 so that maintain high starch production under multiple abiotic stresses. CRISPR/Cas9 allows the introduction of novel alleles or any other novel genetic material (Montagu, 2019), especially by exploiting the genetic diversity of wild plants. For instance, the transformation of the elite maize inbred line DH4866 with the *betA* gene (encoding choline dehydrogenase) from *Escherichia coli* resulted in increased drought tolerance and grain yield in transgenic plants under drought conditions (Quan et al. 2004).

Finally, in response to climate change and population growth, creating climate change-ready crops without a yield penalty is vital for ensuring yield stability. This requires editing or introducing two or more high-quality genes into the elite crop lines to maintain stable starch synthesis under drought and heat stresses.

## Conclusions and future perspectives

Climate change detrimentally affects starch biosynthesis in food crops. Creating climate-resilient crop cultivars with stable starch production under various stress conditions is indispensable for human survival in the future. Improving starch yield and quality as well as crop resistance is of great significance for the development of sustainable agriculture.

CRISPR/Cas9 has demonstrated significant potential for the production of edited crops with large variations in amylose content and starches with unique functionalities. Modifying starch biosynthesis by increasing insusceptibility of starch biosynthetic enzymes to drought an heat stress would directly enhance sink (e.g., developing grains) capacity of starch accumulation in a constantly changing climate, which is important for global food security. Moreover, fine-tuning amylose content to an appropriate level through CRISPR/Cas9 helps to maintain a balance between starch yield and quality. Besides, CRISPR/Cas9 editing crops produce starch that does not require or rarely requires post-harvest processing to alter starch properties, thus beneficial for industry and the environment.

CRISPR/Cas9 can modify genes in highly heterozygous crops to generate transgene-free progeny with stable homozygous traits. The absence of foreign DNA integration in these gene-edited crops is likely to facilitate greater public acceptance in the future. For the elimination of transgenes, selfing is commonly employed in many plant species, including maize. However, in species with that vegetative reproduction such as potato, selfing is not an ideal approach due to self-incompatibility issues in commercial potato cultivars (Bánfalvi et al. 2020; Chincinska et al. 2022). A novel nanoparticle-mediated co-delivery system, which enables the delivery of reagents without integrating them into the genome, represents a promising technology for potato improvement (Li et al. 2023b).

Despite numerous studies on CRISPR/Cas9 editing starch biosynthesis in crops, there are rare reports on improving both starch traits and crop stress tolerance to date. Starch content and quality are controlled by multiple genes, especially in polyploid species such as wheat and sweet potato. Moreover, there are often undesired tradeoffs between crop yield and abiotic stress resistance (Tian et al. 2021), which pose limitations to CRISPR’s ability to create high-quality starch crops that can adapt to climate change. To accelerate the development and agricultural application of such gene-edited crops, future studies should focus on the following areas:

(i) *Characterization of more genes related to starch synthesis, especially those also related to abiotic stress tolerance.* Starch biosynthesis is a complex process controlled by multiple genes and significantly influenced by abiotic stress. The presence of multiple allelic forms of core enzymes indicates the complex regulation of starch biosynthesis. Many of these genes require functional characterization, especially the role of SSRG in RS formation. Hopefully, novel genes that regulate starch biosynthesis and traits can be identified by a combination of conventional genetic analyses and the mapping method (e.g., Nakata et al. 2018). CRISPR/Cas9 targeting of these regulatory and structural genes, as well as *cis*-regulatory sequences, provides an efficient approach for developing crop varieties resistant to adverse climate conditions.
(ii) *Regulatory mechanisms of starch biosynthetic enzymes under climate change.* There is less knowledge about the transcriptional regulation of SSRG in crops, and only a few TFs that regulate starch biosynthesis have been identified (Table 1). Most starch-biosynthetic enzymes are unstable under abiotic stress. The regulatory mechanisms governing starch biosynthetic enzymes in crops still need to be clarified, particularly under climate change conditions. Environmental stress can alter the activity of these enzymes at the transcriptional and post-transcriptional levels, adversely affecting crop yield. Fundamental research is particularly necessary for enzymes that are sensitive to drought and heat stress, such as AGPase, SS, and GBSS. It is necessary to systematically analyze the regulation of starch biosynthetic enzyme activity in crops with improved starch traits, initially under single-stress conditions, such as drought and heat, and subsequently under combinatorial stresses.
(iii) *Field evaluation of the performance of CRISPR/Cas9-edited crops.* Although the knockout of the target genes is useful for functional studies in the lab and could improve certain traits of crops, it potentially causes undesirable phenotypes. CRISPR/Cas9 editing may induce mutations in unrelated genes due to faulty design or close resemblance to the target genes, or it may impact pleiotropic alleles controlling other traits (Naeem et al. 2020). This highlights the need for field evaluation of these CRISPR/Cas9-edited crops for climate resilience. To our knowledge, there are no reports on the stress tolerance evaluation of edited crops with improved starch traits. Furthermore, manipulation of single gene expression has often yielded variable increases in crop yield and unexpected results, suggesting the presence of more complex control mechanisms. For example, in barley, editing *SSIIa* not only increases amylose starch content but also causes profound changes in the transcriptome and metabolome (Yang et al. 2022). Therefore, comprehensive field trials are necessary to evaluate the potential negative effects of modifying starch synthetic enzymes on plant growth, particularly regarding plant adaptation to climate change. Successful field performance is a prerequisite for the widespread adoption of gene-edited crops in agriculture in response to the challenge of a changing climate.

## Data Availability

No data was used for the research described in the article.

## Abbreviations

AGPase: ADP-glucose pyrophosphorylase
APX: Ascorbate peroxidase
DBE: Starch debranching enzyme
DP: Degree of polymerization
GBSS: Granule-bound starch synthase
GWD1: Glucan, water dikinase 1
ISA: Isoamylase
PHO: Starch phosphorylase
*PTST1*: *PROTEIN TARGETING TO STARCH* *1*
PUL: Pullulanase
ROS: Reactive oxygen species
RS: Resistant starch
SBE: Starch branching enzyme
SG: Starch granules
SS: Starch synthase
SSRG: Starch synthesis–related genes
SOD: Superoxide dismutase
TF: Transcription factors

## References

[CR1] Andersson M, Turesson H, Olsson N, Fält AS, Ohlsson P, Gonzalez MN, Samuelsson M, Hofvander P (2018) Genome editing in potato via CRISPR-Cas9 ribonucleoprotein delivery. Physiol Plant 164(4):378–384. 10.1111/ppl.12731 29572864 10.1111/ppl.12731

[CR2] Anwar A, Kim Jk (2020) Transgenic breeding approaches for improving abiotic stress tolerance: recent progress and future perspectives. Int J Mol Sci 21(8):2695. 10.3390/ijms21082695 32295026 10.3390/ijms21082695 PMC7216248

[CR3] Bánfalvi Z, Csákvári E, Villányi V, Kondrák M (2020) Generation of transgene-free PDS mutants in potato by *Agrobacterium*-mediated transformation. BMC Biotechnol 20(1):25. 10.1186/s12896-020-00621-2 32398038 10.1186/s12896-020-00621-2 PMC7216596

[CR4] Ben Mariem S, Soba D, Zhou B, Loladze I, Morales F, Aranjuelo I (2021) Climate change, crop yields, and grain quality of C_3_ cereals: a meta-analysis of CO_2_, temperature, and drought effects. Plants 10(6):1052. 10.3390/plants10061052 34074065 10.3390/plants10061052 PMC8225050

[CR5] Borrill P, Fahy B, Smith AM, Uauy C (2015) Wheat grain filling is limited by grain filling capacity rather than the duration of flag leaf photosynthesis: a case study using NAM RNAi plants. PLoS One 10(8):e0134947. 10.1371/journal.pone.0134947 26241955 10.1371/journal.pone.0134947 PMC4524614

[CR6] Bouzroud S, Gasparini K, Hu G, Barbosa MAM, Rosa BL, Fahr M, Bendaou N, Bouzayen M, Zsögön A, Smouni A, Zouine M (2020) Down regulation and loss of auxin response factor 4 function using CRISPR/Cas9 alters plant growth, stomatal function and improves tomato tolerance to salinity and osmotic stress. Genes 11(3):272. 10.3390/genes11030272 32138192 10.3390/genes11030272 PMC7140898

[CR7] Brummell DA, Watson LM, Zhou J, McKenzie MJ, Hallett IC, Simmons L, Carpenter M, Timmerman-Vaughan GM (2015) Overexpression of STARCH BRANCHING ENZYME II increases short-chain branching of amylopectin and alters the physicochemical properties of starch from potato tuber. BMC Biotechnol 15:28. 10.1186/s12896-015-0143-y 25926043 10.1186/s12896-015-0143-y PMC4414359

[CR8] Bull SE, Seung D, Chanez C, Mehta D, Kuon JE, Truernit E, Hochmuth A, Zurkirchen I, Zeeman SC, Gruissem W, Vanderschuren H (2018) Accelerated ex situ breeding of GBSS- and PTST1-edited cassava for modified starch. Sci Adv 4(9):eaat6086. 10.1126/sciadv.aat6086 30191180 10.1126/sciadv.aat6086 PMC6124905

[CR9] Cao R, Zhao S, Jiao G, Duan Y, Ma L, Dong N, Lu F, Zhu M, Shao G, Hu S, Sheng Z, Zhang J, Tang S, Wei X, Hu P (2022) Opaque3, encoding a transmembrane bZIP transcription factor, regulates endosperm storage protein and starch biosynthesis in rice. Plant Commun 3(6):100463. 10.1016/j.xplc.2022.100463 36258666 10.1016/j.xplc.2022.100463 PMC9700205

[CR10] Chao S, Cai Y, Feng B, Jiao G, Sheng Z, Luo J, Tang S, Wang J, Hu P, Wei X (2019) Editing of rice isoamylase gene *ISA1* provides insights into its function in starch formation. Rice Sci 26(2):77–87. 10.1016/j.rsci.2018.07.001

[CR11] Chen J, Hawkins E, Seung D (2021) Towards targeted starch modification in plants. Curr Opin Plant Biol 60:102013. 10.1016/j.pbi.2021.102013 33677239 10.1016/j.pbi.2021.102013

[CR12] Chen K, Wang Y, Zhang R, Zhang H, Gao C (2019) CRISPR/Cas genome editing and precision plant breeding in agriculture. Annu Rev Plant Biol 70:667–697. 10.1146/annurev-arplant-050718-100049 30835493 10.1146/annurev-arplant-050718-100049

[CR13] Chia T, Chirico M, King R, Ramirez-Gonzalez R, Saccomanno B, Seung D, Simmonds J, Trick M, Uauy C, Verhoeven T, Trafford K (2020) A carbohydrate-binding protein, B-GRANULE CONTENT 1, influences starch granule size distribution in a dose-dependent manner in polyploid wheat. J Exp Bot 71(1):105–115. 10.1093/jxb/erz405 31633795 10.1093/jxb/erz405

[CR14] Chincinska IA, Miklaszewska M, Sołtys-Kalina D (2022) Recent advances and challenges in potato improvement using CRISPR/Cas genome editing. Planta 257(1):25. 10.1007/s00425-022-04054-3 36562862 10.1007/s00425-022-04054-3 PMC9789015

[CR15] Cuesta-Seijo JA, De Porcellinis AJ, Valente AHR, Striebeck A, Voss C, Marri L, Hansson A, Jansson AM, Dinesen MH, Fangel JU, Harholt J, Popovic M, Thieme M, Hochmuth A, Zeeman SC, Mikkelsen TNR, Jï Rgensen RB, Roitsch TG, Mï Ller BL, Braumann I (2019) Amylopectin chain length dynamics and activity signatures of key carbon metabolic enzymes highlight early maturation as culprit for yield reduction of barley endosperm starch after heat stress. Plant Cell Physiol 60(12):2692–2706. 10.1093/pcp/pcz155 31397873 10.1093/pcp/pcz155 PMC6896705

[CR16] Del Mar Martínez-Prada M, Curtin SJ, Gutiérrez-González JJ (2021) Potato improvement through genetic engineering. GM Crops Food 12(1):479–496. 10.1080/21645698.2021.1993688 34991415 10.1080/21645698.2021.1993688 PMC9208627

[CR17] Dong Q, Wang F, Kong J, Xu Q, Li T, Chen L, Chen H, Jiang H, Li C, Cheng B (2019) Functional analysis of ZmMADS1a reveals its role in regulating starch biosynthesis in maize endosperm. Sci Rep 9(1):3253. 10.1038/s41598-019-39612-5 30824731 10.1038/s41598-019-39612-5 PMC6397188

[CR18] Dong X, Zhang D, Liu J, Liu QQ, Liu H, Tian L, Jiang L, le Qu Q (2015) Plastidial disproportionating enzyme participates in starch synthesis in rice endosperm by transferring maltooligosyl groups from amylose and amylopectin to amylopectin. Plant Physiol 169(4):2496–2512. 10.1104/pp.15.01411 26471894 10.1104/pp.15.01411 PMC4677918

[CR19] Fatima Z, Ahmed M, Hussain M, Abbas G, Ul-Allah S, Ahmad S, Ahmed N, Ali MA, Sarwar G, Haque EU, Iqbal P, Hussain S (2020) The fingerprints of climate warming on cereal crops phenology and adaptation options. Sci Rep 10(1):18013. 10.1038/s41598-020-74740-3 33093541 10.1038/s41598-020-74740-3 PMC7581754

[CR20] Figueroa CM, Asencion Diez MD, Ballicora MA, Iglesias AA (2022) Structure, function, and evolution of plant ADP-glucose pyrophosphorylase. Plant Mol Biol 108(4–5):307–323. 10.1007/s11103-021-01235-8 35006475 10.1007/s11103-021-01235-8

[CR21] Fu Y, Hua Y, Luo T, Liu C, Zhang B, Zhang X, Liu Y, Liu Y, Zhu Z, Tao Y, Zhu Z, Li P, Zhu J (2023) Generating waxy rice starch with target type of amylopectin fine structure and gelatinization temperature by waxy gene editing. Carbohydr Polym 306:120595. 10.1016/j.carbpol.2023.120595 36746586 10.1016/j.carbpol.2023.120595

[CR22] Fu Y, Luo T, Hua Y, Yan X, Liu X, Liu Y, Zhang B, Liu R, Zhu Z, Zhu J (2022) Assessment of the characteristics of waxy rice mutants generated by CRISPR/Cas9. Front Plant Sci 13:881964. 10.3389/fpls.2022.881964 35755680 10.3389/fpls.2022.881964 PMC9226628

[CR23] Fujita N, Yoshida M, Asakura N, Ohdan T, Miyao A, Hirochika H, Nakamura Y (2006) Function and characterization of starch synthase I using mutants in rice. Plant Physiol 140(3):1070–1084. 10.1104/pp.105.071845 16443699 10.1104/pp.105.071845 PMC1400558

[CR24] Gao Y, An K, Guo W, Chen Y, Zhang R, Zhang X, Chang S, Rossi V, Jin F, Cao X, Xin M, Peng H, Hu Z, Guo W, Du J, Ni Z, Sun Q, Yao Y (2021) The endosperm-specific transcription factor TaNAC019 regulates glutenin and starch accumulation and its elite allele improves wheat grain quality. Plant Cell 33(3):603–622. 10.1093/plcell/koaa040 33955492 10.1093/plcell/koaa040 PMC8136912

[CR25] Godfray HC, Beddington JR, Crute IR, Haddad L, Lawrence D, Muir JF, Pretty J, Robinson S, Thomas SM, Toulmin C (2010) Food security: the challenge of feeding 9 billion people. Science 327(5967):812–188. 10.1126/science.1185383 20110467 10.1126/science.1185383

[CR26] Goswami S, Kumar RR, Bakshi S, Praveen S (2022) Starch metabolism under heat stress. In: Kumar RR, Praveen S, Rai GK (eds) Thermotolerance in Crop Plants. Springer, Singapore. 10.1007/978-981-19-3800-9_9

[CR27] Guo K, Liang W, Wang S, Guo D, Liu F, Persson S, Herburger K, Petersen BL, Liu X, Blennow A, Zhong Y (2023) Strategies for starch customization: agricultural modification. Carbohydr Polym 321:121336. 10.1016/j.carbpol.2023.121336 10.1016/j.carbpol.2023.121336 37739487

[CR28] He S, Hao X, Wang S, Zhou W, Ma Q, Lu X, Chen L, Zhang P (2022) Starch synthase II plays a crucial role in starch biosynthesis and the formation of multienzyme complexes in cassava storage roots. J Exp Bot 73(8):2540–2557. 10.1093/jxb/erac022 35134892 10.1093/jxb/erac022

[CR29] Huang L, Gu Z, Chen Z, Yu J, Chu R, Tan H, Zhao D, Fan X, Zhang C, Li Q, Liu Q (2021a) Improving rice eating and cooking quality by coordinated expression of the major starch synthesis-related genes, *SSII* and *Wx*, in endosperm. Plant Mol Biol 106(4–5):419–432. 10.1007/s11103-021-01162-8 34129189 10.1007/s11103-021-01162-8

[CR30] Huang L, Li Q, Zhang C, Chu R, Gu Z, Tan H, Zhao D, Fan X, Liu Q (2020) Creating novel *Wx* alleles with fine-tuned amylose levels and improved grain quality in rice by promoter editing using CRISPR/Cas9 system. Plant Biotechnol J 18(11):2164–2166. 10.1111/pbi.13391 32339389 10.1111/pbi.13391 PMC7589223

[CR31] Huang L, Liu Q (2023) High-resistant starch crops for human health. Proc Natl Acad Sci U S A 120(22):e2305990120. 10.1073/pnas.2305990120 37216520 10.1073/pnas.2305990120 PMC10235962

[CR32] Huang L, Tan H, Zhang C, Li Q, Liu Q (2021) Starch biosynthesis in cereal endosperms: an updated review over the last decade. Plant Commun 2(5):100237. 10.1016/j.xplc.2021.100237 34746765 10.1016/j.xplc.2021.100237 PMC8554040

[CR33] Huang L, Xiao Y, Zhao W, Rao Y, Shen H, Gu Z, Fan X, Li Q, Zhang C, Liu Q (2024) Creating high-resistant starch rice by simultaneous editing of SS3a and SS3b. Plant Biotechnol J 22(4):787–789. 10.1111/pbi.14053 37128176 10.1111/pbi.14053 PMC10955483

[CR34] Jung KH, An G, Ronald PC (2008) Towards a better bowl of rice: assigning function to tens of thousands of rice genes. Nat Rev Genet 9:91–101. 10.1038/nrg2286 18160965 10.1038/nrg2286

[CR35] Kang X, Gao W, Cui B, Abd El-Aty AM (2023) Structure and genetic regulation of starch formation in sorghum (*Sorghum bicolor* (L.) Moench) endosperm: a review. Int J Biol Macromol 239:124315. 10.1016/j.ijbiomac.2023.124315 37023877 10.1016/j.ijbiomac.2023.124315

[CR36] Kim KH, Kim JY (2021) Understanding wheat starch metabolism in properties, environmental stress condition, and molecular approaches for value-added utilization. Plants 10(11):2282. 10.3390/plants10112282 34834645 10.3390/plants10112282 PMC8624758

[CR37] Kumar P, Mishra A, Sharma H, Sharma D, Rahim MS, Sharma M, Parveen A, Jain P, Verma SK, Rishi V, Roy J (2018) Pivotal role of bZIPs in amylose biosynthesis by genome survey and transcriptome analysis in wheat (*Triticum aestivum* L.) mutants. Sci Rep 8(1):17240. 10.1038/s41598-018-35366-8 30467374 10.1038/s41598-018-35366-8 PMC6250691

[CR38] Lafiandra D, Sestili F, Sissons M, Kiszonas A, Morris CF (2022) Increasing the versatility of durum wheat through modifications of protein and starch composition and grain hardness. Foods 11(11):1532. 10.3390/foods11111532 35681282 10.3390/foods11111532 PMC9180912

[CR39] Lesk C, Coffel E, Winter J, Ray D, Zscheischler J, Seneviratne SI, Horton R (2021) Stronger temperature-moisture couplings exacerbate the impact of climate warming on global crop yields. Nat Food 2(9):683–691. 10.1038/s43016-021-00341-6 37117467 10.1038/s43016-021-00341-6

[CR40] Li J, Jiao G, Sun Y, Chen J, Zhong Y, Yan L, Jiang D, Ma Y, Xia L (2021) Modification of starch composition, structure and properties through editing of *TaSBEIIa* in both winter and spring wheat varieties by CRISPR/Cas9. Plant Biotechnol J 19(5):937–951. 10.1111/pbi.13519 33236499 10.1111/pbi.13519 PMC8131058

[CR41] Li M, Daygon VD, Solah V, Dhital S (2023a) Starch granule size: does it matter? Crit Rev Food Sci Nutr 63(19):3683–3703. 10.1080/10408398.2021.1992607 34704861 10.1080/10408398.2021.1992607

[CR42] Li M, Sun X, Yin M, Shen J, Yan S (2023b) Recent advances in nanoparticle-mediated co-delivery system: a promising strategy in medical and agricultural field. Int J Mol Sci 24(6):5121. 10.3390/ijms24065121 36982200 10.3390/ijms24065121 PMC10048901

[CR43] Li Y, Jiang Y, Cao D, Dang B, Yang X, Fan S, Shen Y, Li G, Liu B (2024) Creating a zero amylose barley with high soluble sugar content by genome editing. Plant Mol Biol 114(3):50. 10.1007/s11103-024-01445-w 38656412 10.1007/s11103-024-01445-w

[CR44] Linebarger CR, Boehlein SK, Sewell AK, Shaw J, Hannah LC (2005) Heat stability of maize endosperm ADP-glucose pyrophosphorylase is enhanced by insertion of a cysteine in the N terminus of the small subunit. Plant Physiol 139(4):1625–1634. 10.1104/pp.105.067637 16299180 10.1104/pp.105.067637 PMC1310547

[CR45] Liu J, Wu MW, Liu CM (2022a) Cereal endosperms: development and storage product accumulation. Annu Rev Plant Biol 73:255–291. 10.1146/annurev-arplant-070221-024405 35226815 10.1146/annurev-arplant-070221-024405

[CR46] Liu K, Wang X, Liu H, Wu J, Liang F, Li S, Zhang J, Zhang J, Peng X (2022b) OsAT1, an anion transporter, negatively regulates grain size and yield in rice. Physiol Plant 174(3):e13692. 10.1111/ppl.13692 35482934 10.1111/ppl.13692

[CR47] Liu S, Shao G, Jiao G, Zhu M, Wu J, Cao R, Chen Y, Xie L, Sheng Z, Tang S (2021) Editing of rice endosperm plastidial phosphorylase gene *OsPho1* advances its function in starch synthesis. Rice Sci 28(3):209–211. 10.1016/j.rsci.2020.10.001

[CR48] Liu Y, Hou J, Wang X, Li T, Majeed U, Hao C, Zhang X (2020) The NAC transcription factor NAC019-A1 is a negative regulator of starch synthesis in wheat developing endosperm. J Exp Bot 71(19):5794–5807. 10.1093/jxb/eraa333 32803271 10.1093/jxb/eraa333

[CR49] Liu Z, Jiang S, Jiang L, Li W, Tang Y, He W, Zhang X (2022c) Transcription factor OsSGL is a regulator of starch synthesis and grain quality in rice. J Exp Bot 73(11):3417–3430. 10.1093/jxb/erac068 35182423 10.1093/jxb/erac068

[CR50] Lu D, Shen X, Cai X, Yan F, Lu W, Shi YC (2014) Effects of heat stress during grain filling on the structure and thermal properties of waxy maize starch. Food Chem 143:313–318. 10.1016/j.foodchem.2013.07.089 24054245 10.1016/j.foodchem.2013.07.089

[CR51] Lu H, Hu Y, Wang C, Liu W, Ma G, Han Q, Ma D (2019) Effects of high temperature and drought stress on the expression of gene encoding enzymes and the activity of key enzymes involved in starch biosynthesis in wheat grains. Front Plant Sci 10:1414. 10.3389/fpls.2019.01414 31798603 10.3389/fpls.2019.01414 PMC6863091

[CR52] Luo S, Ma Q, Zhong Y, Jing J, Wei Z, Zhou W, Lu X, Tian Y, Zhang P (2022) Editing of the starch branching enzyme gene SBE2 generates high-amylose storage roots in cassava. Plant Mol Biol 108(4–5):429–442. 10.1007/s11103-021-01215-y 34792751 10.1007/s11103-021-01215-y

[CR53] Lyu R, Ahmed S, Fan W, Yang J, Wu X, Zhou W, Zhang P, Yuan L, Wang H (2021) Engineering properties of sweet potato starch for industrial applications by biotechnological techniques including genome editing. Int J Mol Sci 22(17):9533. 10.3390/ijms22179533 34502441 10.3390/ijms22179533 PMC8431112

[CR54] Madhawan A, Sharma A, Bhandawat A, Rahim MS, Kumar P, Mishra A, Parveen A, Sharma H, Verma SK, Roy J (2020) Identification and characterization of long non-coding RNAs regulating resistant starch biosynthesis in bread wheat (*Triticum aestivum* L.). Genomics 2020(5):3065–3074. 10.1016/j.ygeno.2020.05.014 10.1016/j.ygeno.2020.05.014 32447006

[CR55] Malinova I, Alseekh S, Feil R, Fernie AR, Baumann O, Schöttler MA, Lunn JE, Fettke J (2017) Starch synthase 4 and plastidal phosphorylase differentially affect starch granule number and morphology. Plant Physiol 174(1):73–85. 10.1104/pp.16.01859 28275148 10.1104/pp.16.01859 PMC5411139

[CR56] Mao H, Jiang C, Tang C, Nie X, Du L, Liu Y, Cheng P, Wu Y, Liu H, Kang Z, Wang X (2023) Wheat adaptation to environmental stresses under climate change: molecular basis and genetic improvement. Mol Plant 16(10):1564–1589. 10.1016/j.molp.2023.09.001 37671604 10.1016/j.molp.2023.09.001

[CR57] Montagu MV (2019) The future of plant biotechnology in a globalized and environmentally endangered world. Genet Mol Biol 43(1 suppl 2):e20190040. 10.1590/1678-4685-GMB-2019-0040 31930275 10.1590/1678-4685-GMB-2019-0040 PMC7216575

[CR58] Naeem M, Majeed S, Hoque MZ, Ahmad I (2020) Latest developed strategies to minimize the off-target effects in CRISPR-Cas-mediated genome editing. Cells 9(7):1608. 10.3390/cells9071608 32630835 10.3390/cells9071608 PMC7407193

[CR59] Nakata M, Miyashita T, Kimura R, Nakata Y, Takagi H, Kuroda M, Yamaguchi T, Umemoto T, Yamakawa H (2018) Mutmapplus identified novel mutant alleles of a rice *starch branching enzyme IIb* gene for fine-tuning of cooked rice texture. Plant Biotechnol J 16(1):111–123. 10.1111/pbi.12753 28499068 10.1111/pbi.12753 PMC5785365

[CR60] Niu L, Ding H, Hao R, Liu H, Wu X, Hu X, Wang W (2019a) A rapid and universal method for isolating starch granules in plant tissues. Plant Cell Environ 42(12):3355–3371. 10.1111/pce.13631 31429107 10.1111/pce.13631

[CR61] Niu L, Ding H, Zhang J, Wang W (2019b) Proteomic analysis of starch biosynthesis in maize seeds. Starch 71:1800294. 10.1002/star.201800294

[CR62] Niu L, Liu L, Zhang J, Scali M, Wang W, Hu X, Wu X (2023) Genetic engineering of starch biosynthesis in maize seeds for efficient enzymatic digestion of starch during bioethanol production. Int J Mol Sci 24(4):3927. 10.3390/ijms24043927 36835340 10.3390/ijms24043927 PMC9967003

[CR63] Niu L, Wu X, Liu H, Hu X, Wang W (2024) Leaf starch degradation by β-amylase ZmBAM8 influences drought tolerance in maize. Carbohydr Polym 345:122555. 10.1016/j.carbpol.2024.122555 39227118 10.1016/j.carbpol.2024.122555

[CR64] Parveen A, Rahim MS, Sharma A, Mishra A, Kumar P, Fandade V, Kumar P, Bhandawat A, Verma SK, Roy J (2021) Genome-wide analysis of RING-type E3 ligase family identifies potential candidates regulating high amylose starch biosynthesis in wheat (*Triticum aestivum* L.). Sci Rep 11(1):11461. 10.1038/s41598-021-90685-7 34075092 10.1038/s41598-021-90685-7 PMC8169666

[CR65] Peng C, Wang Y, Liu F, Ren Y, Zhou K, Lv J, Zheng M, Zhao S, Zhang L, Wang C, Jiang L, Zhang X, Guo X, Bao Y, Wan J (2014) Floury endosperm6 encodes a CBM48 domain-containing protein involved in compound granule formation and starch synthesis in rice endosperm. Plant J 77(6):917–930. 10.1111/tpj.12444 24456533 10.1111/tpj.12444

[CR66] Pérez L, Soto E, Farré G, Juanos J, Villorbina G, Bassie L, Medina V, Serrato AJ, Sahrawy M, Rojas JA, Romagosa I, Muñoz P, Zhu C, Christou P (2019) CRISPR/Cas9 mutations in the rice waxy/GBSSI gene induce allele-specific and zygosity-dependent feedback effects on endosperm starch biosynthesis. Plant Cell Rep 38(3):417–433. 10.1007/s00299-019-02388-z 30715580 10.1007/s00299-019-02388-z

[CR67] Pfotenhauer AC, Occhialini A, Harbison SA, Li L, Piatek AA, Luckett CR, Yang Y, Stewart CN Jr, Lenaghan SC (2023) Genome-editing of *FtsZ1* for alteration of starch granule size in potato tubers. Plants 12(9):1878. 10.3390/plants12091878 37176936 10.3390/plants12091878 PMC10180631

[CR68] Priya BNV, Arun Pandiyan I, Reddy TV, Vinay K, Amarnath M, Chidanand U, Hemanth V, Saiprasad GVS (2021) Identification of SNPs in crucial starch biosynthesis genes in rice. J Genet 100:8. 10.1007/s12041-020-01251-5 33707359

[CR69] Qi X, Wu H, Jiang H, Zhu J, Huang C, Zhang X, Liu C, Cheng B (2020) Conversion of a normal maize hybrid into a waxy version using in vivo CRISPR/Cas9 targeted mutation activity. Crop J 8:440–448. 10.1016/j.cj.2020.01.006

[CR70] Qu J, Xu S, Zhang Z, Chen G, Zhong Y, Liu L, Zhang R, Xue J, Guo D (2018) Evolutionary, structural and expression analysis of core genes involved in starch synthesis. Sci Rep 8(1):12736. 10.1038/s41598-018-30411-y 30143668 10.1038/s41598-018-30411-y PMC6109180

[CR71] Quan R, Shang M, Zhang H, Zhao Y, Zhang J (2004) Engineering of enhanced glycine betaine synthesis improves drought tolerance in maize. Plant Biotechnol J 2(6):477–486. 10.1111/j.1467-7652.2004.00093.x 17147620 10.1111/j.1467-7652.2004.00093.x

[CR72] Razzaq MK, Aleem M, Mansoor S, Khan MA, Rauf S, Iqbal S, Siddique KHM (2021) Omics and CRISPR-Cas9 approaches for molecular insight, functional gene analysis, and stress tolerance development in crops. Int J Mol Sci 22(3):1292. 10.3390/ijms22031292 33525517 10.3390/ijms22031292 PMC7866018

[CR73] Ren Y, Sun X, Nie J, Guo P, Wu X, Zhang Y, Gao M, Niaz M, Yang X, Sun C, Zhang N, Chen F (2023) Mapping QTL conferring flag leaf senescence in durum wheat cultivars. Mol Breed 43(8):66. 10.1007/s11032-023-01410-3 37564974 10.1007/s11032-023-01410-3 PMC10409934

[CR74] Rezaei EE, Webber H, Asseng S, Boote K, Durand JL, Ewert F, Martre P, MacCarthy DS (2023) Climate change impacts on crop yields. Nat Rev Earth Environ 4:831–846. 10.1038/s43017-023-00491-0

[CR75] Ribeiro C, Hennen-Bierwagen TA, Myers AM, Cline K, Settles AM (2020) Engineering 6-phosphogluconate dehydrogenase improves grain yield in heat-stressed maize. Proc Natl Acad Sci U S A 117(52):33177–33185. 10.1073/pnas.2010179117 33323483 10.1073/pnas.2010179117 PMC7776907

[CR76] Saito M, Tanaka T, Sato K, Vrinten P, Nakamura T (2018) A single nucleotide polymorphism in the “Fra” gene results in fractured starch granules in barley. Theor Appl Genet 131(2):353–364. 10.1007/s00122-017-3006-1 29098311 10.1007/s00122-017-3006-1

[CR77] Samset BH, Zhou C, Fuglestvedt JS, Lund MT, Marotzke J, Zelinka MD (2023) Steady global surface warming from 1973 to 2022 but increased warming rate after 1990. Commun Earth Environ 4:400. 10.1038/s43247-023-01061-4

[CR78] Sánchez-León S, Gil-Humanes J, Ozuna CV, Giménez MJ, Sousa C, Voytas DF, Barro F (2018) Low-gluten, nontransgenic wheat engineered with CRISPR/Cas9. Plant Biotechnol J 16(4):902–910. 10.1111/pbi.12837 28921815 10.1111/pbi.12837 PMC5867031

[CR79] Senapati N, Stratonovitch P, Paul MJ, Semenov MA (2019) Drought tolerance during reproductive development is important for increasing wheat yield potential under climate change in Europe. J Exp Bot 70(9):2549–2560. 10.1093/jxb/ery226 29901813 10.1093/jxb/ery226 PMC6487587

[CR80] Shafi A, Pal AK, Sharma V, Kalia S, Kumar S, Ahuja PS, Singh AK (2017) Transgenic potato plants overexpressing SOD and APX exhibit enhanced lignification and starch biosynthesis with improved salt stress tolerance. Plant Mol Biol Rep 35:504–518. 10.1007/s11105-017-1041-3

[CR81] Shan Q, Wang Y, Li J, Zhang Y, Chen K, Liang Z, Zhang K, Liu J, Xi JJ, Qiu JL, Gao C (2013) Targeted genome modification of crop plants using a CRISPR-Cas system. Nat Biotechnol 31(8):686–688. 10.1038/nbt.2650 23929338 10.1038/nbt.2650

[CR82] Shen L, Li J, Li Y (2022) Resistant starch formation in rice: genetic regulation and beyond. Plant Communications 3(3):100329. 10.1016/j.xplc.2022.100329 35576157 10.1016/j.xplc.2022.100329 PMC9251435

[CR83] Smith AM, Zeeman SC (2020) Starch: a flexible, adaptable carbon store coupled to plant growth. Annu Rev Plant Biol 71:217–245. 10.1146/annurev-arplant-050718-100241 32075407 10.1146/annurev-arplant-050718-100241

[CR84] Song Y, Luo G, Shen L, Yu K, Yang W, Li X, Sun J, Zhan K, Cui D, Liu D, Zhang A (2020) TubZIP28, a novel bZIP family transcription factor from *Triticum urartu*, and TabZIP28, its homologue from *Triticum aestivum*, enhance starch synthesis in wheat. New Phytol 226(5):1384–1398. 10.1111/nph.16435 31955424 10.1111/nph.16435

[CR85] Steinwand MA, Ronald PC (2020) Crop biotechnology and the future of food. Nat Food 1:273–283. 10.1038/s43016-020-0072-3

[CR86] Sun Y, Jiao G, Liu Z, Zhang X, Li J, Guo X, Du W, Du J, Francis F, Zhao Y, Xia L (2017) Generation of high-amylose rice through CRISPR/Cas9-mediated targeted mutagenesis of starch branching enzymes. Front Plant Sci 8:298. 10.3389/fpls.2017.00298 28326091 10.3389/fpls.2017.00298 PMC5339335

[CR87] Takeuchi A, Ohnuma M, Teramura H, Asano K, Noda T, Kusano H, Tamura K, Shimada H (2021) Creation of a potato mutant lacking the starch branching enzyme gene StSBE3 that was generated by genome editing using the CRISPR/dMac3-Cas9 system. Plant Biotechnol 38(3):345–353. 10.5511/plantbiotechnology.21.0727a 10.5511/plantbiotechnology.21.0727a PMC8562579 34782822

[CR88] Tappiban P, Ying Y, Xu F, Bao J (2021) Proteomics and post-translational modifications of starch biosynthesis-related proteins in developing seeds of rice. Int J Mol Sci 22(11):5901. 10.3390/ijms22115901 34072759 10.3390/ijms22115901 PMC8199009

[CR89] Tian Z, Wang JW, Li J, Han B (2021) Designing future crops: challenges and strategies for sustainable agriculture. Plant J 105(5):1165–1178. 10.1111/tpj.15107 33258137 10.1111/tpj.15107

[CR90] Toinga-Villafuerte S, Vales MI, Awika JM, Rathore KS (2022) CRISPR/Cas9-mediated mutagenesis of the granule-bound starch synthase gene in the potato variety Yukon Gold to obtain amylose-free starch in tubers. Int J Mol Sci 23(9):4640. 10.3390/ijms23094640 35563030 10.3390/ijms23094640 PMC9101600

[CR91] Toyosawa Y, Kawagoe Y, Matsushima R, Crofts N, Ogawa M, Fukuda M, Kumamaru T, Okazaki Y, Kusano M, Saito K, Toyooka K, Sato M, Ai Y, Jane JL, Nakamura Y, Fujita N (2016) Deficiency of starch synthase IIIa and IVb alters starch granule morphology from polyhedral to spherical in rice endosperm. Plant Physiol 170(3):1255–1270. 10.1104/pp.15.01232 26747287 10.1104/pp.15.01232 PMC4775109

[CR92] Tuncel A, Corbin KR, Ahn-Jarvis J, Harris S, Hawkins E, Smedley MA, Harwood W, Warren FJ, Patron NJ, Smith AM (2019) Cas9-mediated mutagenesis of potato starch-branching enzymes generates a range of tuber starch phenotypes. Plant Biotechnol J 17(12):2259–2271. 10.1111/pbi.13137 31033104 10.1111/pbi.13137 PMC6835119

[CR93] Utsumi Y, Utsumi C, Tanaka M, Takahashi S, Okamoto Y, Ono M, Nakamura Y, Seki M (2022) Suppressed expression of starch branching enzyme 1 and 2 increases resistant starch and amylose content and modifies amylopectin structure in cassava. Plant Mol Biol 108(4–5):413–427. 10.1007/s11103-021-01209-w 34767147 10.1007/s11103-021-01209-w

[CR94] Vollen K, Alonso JM, Stepanova AN (2025) Beyond a few bases: methods for large DNA insertion and gene targeting in plants. Plant J 121(6):e70099. 10.1111/tpj.70099 40121601 10.1111/tpj.70099 PMC11930290

[CR95] Wang A, Jing Y, Cheng Q, Zhou H, Wang L, Gong W, Kou L, Liu G, Meng X, Chen M, Ma H, Shu X, Yu H, Wu D, Li J (2023a) Loss of function of *SSIIIa* and *SSIIIb* coordinately confers high RS content in cooked rice. Proc Natl Acad Sci U S A 120(19):e2220622120. 10.1073/pnas.2220622120 37126676 10.1073/pnas.2220622120 PMC10175802

[CR96] Wang H, Wu Y, Zhang Y, Yang J, Fan W, Zhang H, Zhao S, Yuan L, Zhang P (2019) CRISPR/Cas9-based mutagenesis of starch biosynthetic genes in sweet potato (*Ipomoea batatas*) for the improvement of starch quality. Int J Mol Sci 20(19):4702. 10.3390/ijms20194702 31547486 10.3390/ijms20194702 PMC6801948

[CR97] Wang L, Liu L, Zhao J, Li C, Wu H, Zhao H, Wu Q (2023b) Granule-bound starch synthase in plants: towards an understanding of their evolution, regulatory mechanisms, applications, and perspectives. Plant Sci 336:111843. 10.1016/j.plantsci.2023.111843 37648115 10.1016/j.plantsci.2023.111843

[CR98] Wang L, Wang Y, Makhmoudova A, Nitschke F, Tetlow IJ, Emes MJ (2022) CRISPR-Cas9-mediated editing of starch branching enzymes results in altered starch structure in *Brassica napus*. Plant Physiol 188(4):1866–1886. 10.1093/plphys/kiab535 34850950 10.1093/plphys/kiab535 PMC8968267

[CR99] Wang R, Ren Y, Yan H, Teng X, Zhu X, Wang Y, Zhang X, Guo X, Lin Q, Cheng Z, Lei C, Wang J, Jiang L, Wang Y, Wan J (2021a) Enlarged starch grain1 affects amyloplast development and starch biosynthesis in rice endosperm. Plant Sci 305:110831. 10.1016/j.plantsci.2021.110831 33691965 10.1016/j.plantsci.2021.110831

[CR100] Wang S, Chao C, Cai J, Niu B, Copeland L, Wang S (2020) Starch-lipid and starch-lipid-protein complexes: a comprehensive review. Compr Rev Food Sci Food Saf 19(3):1056–1079. 10.1111/1541-4337.12550 33331685 10.1111/1541-4337.12550

[CR101] Wang X, Hou L, Lu Y, Wu B, Gong X, Liu M, Wang J, Sun Q, Vierling E, Xu S (2018) Metabolic adaptation of wheat grain contributes to a stable filling rate under heat stress. J Exp Bot 69(22):5531–5545. 10.1093/jxb/ery303 30476278 10.1093/jxb/ery303 PMC6255704

[CR102] Wang Z, Wei K, Xiong M, Wang JD, Zhang CQ, Fan XL, Huang LC, Zhao DS, Liu QQ, Li QF (2021b) Glucan, water-dikinase 1 (GWD1), an ideal biotechnological target for potential improving yield and quality in rice. Plant Biotechnol J 19(12):2606–2618. 10.1111/pbi.13686 34416068 10.1111/pbi.13686 PMC8633486

[CR103] Wu J, Chen L, Chen M, Zhou W, Dong Q, Jiang H, Cheng B (2019) The DOF-domain transcription factor ZmDOF36 positively regulates starch synthesis in transgenic maize. Front Plant Sci 10:465. 10.3389/fpls.2019.00465 31031791 10.3389/fpls.2019.00465 PMC6474321

[CR104] Wu W, Qu J, Blennow A, Herburger K, Hebelstrup KH, Guo K (2022) The effects of drought treatments on biosynthesis and structure of maize starches with different amylose content. Carbohydr Polym 297:120045. 10.1016/j.carbpol.2022.120045 36184182 10.1016/j.carbpol.2022.120045

[CR105] Xiao Q, Liu T, Ling M, Ma Q, Cao W, Xing F, Huang T, Zhang Y, Duan H, Liu Z (2022) Genome-wide identification of *DOF* gene family and the mechanism dissection of *SbDof21* regulating starch biosynthesis in sorghum. Int J Mol Sci 23(20):12152. 10.3390/ijms232012152 36293009 10.3390/ijms232012152 PMC9603474

[CR106] Xiong W, Reynolds M, Xu Y (2022) Climate change challenges plant breeding. Curr Opin Plant Biol 70:102308. 10.1016/j.pbi.2022.102308 36279790 10.1016/j.pbi.2022.102308

[CR107] Xu Y, Lin Q, Li X, Wang F, Chen Z, Wang J, Li W, Fan F, Tao Y, Jiang Y, Wei X, Zhang R, Zhu QH, Bu Q, Yang J, Gao C (2021) Fine-tuning the amylose content of rice by precise base editing of the *Wx* gene. Plant Biotechnol J 19(1):11–13. 10.1111/pbi.13433 32558105 10.1111/pbi.13433 PMC7769246

[CR108] Yang H, Dong X, Chai Y, Cui S, Tian L, Zhang J, Qu LQ (2025) Loss-of-function of SSIIa and SSIIIa confers high resistant starch content in rice endosperm. Carbohydr Polym 348(Pt B):122871. 10.1016/j.carbpol.2024.122871 39567160 10.1016/j.carbpol.2024.122871

[CR109] Yang H, Gu X, Ding M, Lu W, Lu D (2018) Heat stress during grain filling affects activities of enzymes involved in grain protein and starch synthesis in waxy maize. Sci Rep 8(1):15665. 10.1038/s41598-018-33644-z 30353095 10.1038/s41598-018-33644-z PMC6199321

[CR110] Yang H, Gu X, Ding M, Lu W, Lu D (2019) Activities of starch synthetic enzymes and contents of endogenous hormones in waxy maize grains subjected to post-silking water deficit. Sci Rep 9(1):7059. 10.1038/s41598-019-43484-0 31065011 10.1038/s41598-019-43484-0 PMC6505039

[CR111] Yang Q, Ding J, Feng X, Zhong X, Lan J, Tang H, Harwood W, Li Z, Guzmán C, Xu Q, Zhang Y, Jiang Y, Qi P, Deng M, Ma J, Wang J, Chen G, Lan X, Wei Y, Zheng Y, Jiang Q (2022) Editing of the *starch synthase IIa* gene led to transcriptomic and metabolomic changes and high amylose starch in barley. Carbohydr Polym 285:119238. 10.1016/j.carbpol.2022.119238 35287861 10.1016/j.carbpol.2022.119238

[CR112] You Y, Zhang M, Yang W, Li C, Liu Y, Li C, He J, Wu W (2020) Starch phosphorylation and the in vivo regulation of starch metabolism and characteristics. Int J Biol Macromol 159:823–831. 10.1016/j.ijbiomac.2020.05.156 32445823 10.1016/j.ijbiomac.2020.05.156

[CR113] Yu G, Mou Y, Shoaib N, He X, Liu L, Di R, Mughal N, Zhang N, Huang Y (2023) Serine 31 phosphorylation-driven regulation of AGPase activity: potential implications for enhanced starch yields in crops. Int J Mol Sci 24(20):15283. 10.3390/ijms242015283 37894964 10.3390/ijms242015283 PMC10607544

[CR114] Yu S, Zhang F, Li C, Gilbert RG (2017) Molecular structural differences between maize leaf and endosperm starches. Carbohydr Polym 161:10–15. 10.1016/j.carbpol.2016.12.064 28189218 10.1016/j.carbpol.2016.12.064

[CR115] Yu W, Wang L, Zhao R, Sheng J, Zhang S, Li R, Shen L (2019) Knockout of *SlMAPK3* enhances tolerance to heat stress involving ROS homeostasis in tomato plants. BMC Plant Biol 19(1):354. 10.1186/s12870-019-1939-z 31412779 10.1186/s12870-019-1939-z PMC6694692

[CR116] Yu X, Li B, Wang L, Chen X, Wang W, Gu Y, Wang Z, Xiong F (2016) Effect of drought stress on the development of endosperm starch granules and the composition and physicochemical properties of starches from soft and hard wheat. J Sci Food Agric 96(8):2746–2754. 10.1002/jsfa.7439 26311190 10.1002/jsfa.7439

[CR117] Zeng D, Liu T, Ma X, Wang B, Zheng Z, Zhang Y, Xie X, Yang B, Zhao Z, Zhu Q, Liu YG (2020) Quantitative regulation of Waxy expression by CRISPR/Cas9-based promoter and 5’UTR-intron editing improves grain quality in rice. Plant Biotechnol J 18(12):2385–2387. 10.1111/pbi.13427 32485068 10.1111/pbi.13427 PMC7680535

[CR118] Zhang C, Zhou L, Zhu Z, Lu H, Zhou X, Qian Y, Li Q, Lu Y, Gu M, Liu Q (2016) Characterization of grain quality and starch fine structure of two Japonica rice (*Oryza sativa*) cultivars with good sensory properties at different temperatures during the filling stage. J Agric Food Chem 64(20):4048–4057. 10.1021/acs.jafc.6b00083 27128366 10.1021/acs.jafc.6b00083

[CR119] Zhang H, Xu H, Feng M, Zhu Y (2018a) Suppression of *OsMADS7* in rice endosperm stabilizes amylose content under high temperature stress. Plant Biotechnol J 16(1):18–26. 10.1111/pbi.12745 28429576 10.1111/pbi.12745 PMC5785353

[CR120] Zhang J, Zhang H, Botella JR, Zhu JK (2018b) Generation of new glutinous rice by CRISPR/Cas9-targeted mutagenesis of the waxy gene in elite rice varieties. J Integr Plant Biol 60(5):369–375. 10.1111/jipb.12620 29210506 10.1111/jipb.12620 PMC5938116

[CR121] Zhang S, Zhang R, Gao J, Song G, Li J, Li W, Qi Y, Li Y, Li G (2021) CRISPR/Cas9-mediated genome editing for wheat grain quality improvement. Plant Biotechnol J 19(9):1684–1686. 10.1111/pbi.13647 34143557 10.1111/pbi.13647 PMC8428824

[CR122] Zhang X, Xie S, Han J, Zhou Y, Liu C, Zhou Z, Wang F, Cheng Z, Zhang J, Hu Y, Hao Z, Li M, Zhang D, Yong H, Huang Y, Weng J, Li X (2019a) Integrated transcriptome, small RNA, and degradome analysis reveals the complex network regulating starch biosynthesis in maize. BMC Genomics 20(1):574. 10.1186/s12864-019-5945-1 31296166 10.1186/s12864-019-5945-1 PMC6625009

[CR123] Zhang Z, Dong J, Ji C, Wu Y, Messing J (2019b) NAC-type transcription factors regulate accumulation of starch and protein in maize seeds. Proc Natl Acad Sci U S A 116(23):11223–11228. 10.1073/pnas.1904995116 31110006 10.1073/pnas.1904995116 PMC6561305

[CR124] Zhang Z, Hu Y, Yu S, Zhao X, Dai G, Deng G, Bao J (2022) Effects of drought stress and elevated CO_2_ on starch fine structures and functional properties in *indica* rice. Carbohydr Polym 297:120044. 10.1016/j.carbpol.2022.120044 36184181 10.1016/j.carbpol.2022.120044

[CR125] Zhang Z, Hua L, Gupta A, Tricoli D, Edwards KJ, Yang B, Li W (2019c) Development of an *Agrobacterium*-delivered CRISPR/Cas9 system for wheat genome editing. Plant Biotechnol J 17(8):1623–1635. 10.1111/pbi.13088 30706614 10.1111/pbi.13088 PMC6662106

[CR126] Zhao K, Tao Y, Liu M, Yang D, Zhu M, Ding J, Zhu X, Guo W, Zhou G, Li C (2022) Does temporary heat stress or low temperature stress similarly affect yield, starch, and protein of winter wheat grain during grain filling? J Cereal Sci 103:103408. 10.1016/j.jcs.2021.103408

[CR127] Zhou H, Liu B, Weeks DP, Spalding MH, Yang B (2014) Large chromosomal deletions and heritable small genetic changes induced by CRISPR/Cas9 in rice. Nucleic Acids Res 42(17):10903–10914. 10.1093/nar/gku806 25200087 10.1093/nar/gku806 PMC4176183

[CR128] Zhou H, Wang L, Liu G, Meng X, Jing Y, Shu X, Kong X, Sun J, Yu H, Smith SM, Wu D, Li J (2016) Critical roles of soluble starch synthase SSIIIa and granule-bound starch synthase Waxy in synthesizing resistant starch in rice. Proc Natl Acad Sci U S A 113(45):12844–12849. 10.1073/pnas.1615104113 27791174 10.1073/pnas.1615104113 PMC5111662

